# Discovery of large genomic inversions using long range information

**DOI:** 10.1186/s12864-016-3444-1

**Published:** 2017-01-10

**Authors:** Marzieh Eslami Rasekh, Giorgia Chiatante, Mattia Miroballo, Joyce Tang, Mario Ventura, Chris T. Amemiya, Evan E. Eichler, Francesca Antonacci, Can Alkan

**Affiliations:** 1 Department of Computer Engineering, Bilkent University, Bilkent, 06800 Ankara Turkey; 2 Department of Biology, University of Bari, Via Orabona 4, 70125 Bari, Italy; 3 Benaroya Research Institute, 1201 Ninth Avenue, 98101 Seattle, WA USA; 4 Department of Genome Sciences and Howard Hughes Medical Institute, University of Washington, 3720 15th Avenue NE, 98195 Seattle, WA USA

**Keywords:** Structural variation, Long range sequencing, Linked-reads, Inversion, Read clouds

## Abstract

**Background:**

Although many algorithms are now available that aim to characterize different classes of structural variation, discovery of balanced rearrangements such as inversions remains an open problem. This is mainly due to the fact that breakpoints of such events typically lie within segmental duplications or common repeats, which reduces the mappability of short reads. The algorithms developed within the 1000 Genomes Project to identify inversions are limited to relatively short inversions, and there are currently no available algorithms to discover large inversions using high throughput sequencing technologies.

**Results:**

Here we propose a novel algorithm, Valor, to discover large inversions using new sequencing methods that provide long range information such as 10X Genomics linked-read sequencing, pooled clone sequencing, or other similar technologies that we commonly refer to as *long range sequencing*. We demonstrate the utility of Valor using both pooled clone sequencing and 10X Genomics linked-read sequencing generated from the genome of an individual from the HapMap project (NA12878). We also provide a comprehensive comparison of Valor against several state-of-the-art structural variation discovery algorithms that use whole genome shotgun sequencing data.

**Conclusions:**

In this paper, we show that Valor is able to accurately discover all previously identified and experimentally validated large inversions in the same genome with a low false discovery rate. Using Valor, we also predicted a novel inversion, which we validated using fluorescent in situ hybridization.

Valor is available at https://github.com/BilkentCompGen/Valor

**Electronic supplementary material:**

The online version of this article (doi:10.1186/s12864-016-3444-1) contains supplementary material, which is available to authorized users.

## Background

Genomic structural variants (SVs) are defined as alterations in the DNA that affect >50 bp that may delete, insert, duplicate, invert, or move genomic sequence [1]. SVs are shown to be common in human genomes [2, 3], which caused increased interest in the characterization of both normal [4–7], and disease-causing large variants [8, 9]. Furthermore, SVs are known to be one of the driving forces of creation of new haplotypes [10] and evolution [11].

A subset of SVs, namely *copy number variations* (CNVs), were initially identified using bacterial artificial chromosome (BAC) and oligo array comparative genomic hybridization (arrayCGH) [2, 3, 12, 13], and SNP genotyping arrays [12, 14]. A more detailed map of SVs was made possible using fosmid end sequencing [4, 5]; however this method was too expensive and time-consuming since it involved creating and plating of fosmid libraries followed by Sanger sequencing. Introduction of high throughput sequencing (HTS) finally made it possible to screen the genomes of many [15–18] to thousands [6, 7, 19] of individuals.

Although there are now many algorithms to discover and genotype SVs using HTS data [1, 20], they mainly focus on CNVs, which change the amount of DNA, such as deletions, duplications, insertions, and retrotranspositions. Other types of SVs, namely *balanced rearrangements* such as inversions and translocations are harder to detect due to the fact that their breakpoints usually lie within complex repeats that reduce mappability. Balanced rearrangements also do not alter the read depth, which makes the use of read depth signature [16, 18, 20] irrelevant for their detection. Therefore, very few attempts have been taken to characterize inversions which are reliable only for small inversions (~10-50 Kbp) [17, 21–23], and exhibit high false discovery rates in translocation call sets [24]. Another algorithm, GASVPro [25] is also able to detect inversions with a size limit up to 500 Kbp; however its sensitivity and specificity for large inversions are yet untested. Characterization of larger genomic inversions using HTS remains an open problem.

### Motivation and approach

Most known examples of large inversions have been identified in studies on human disease where inversions have no detectable effect in parents, but increase the risk of a disease-associated rearrangement in the offspring. In the Williams-Beuren syndrome, for example, 25–30% of transmitting parents carry a 1.5 Mbp inversion encompassing a commonly deleted region, whereas the same inversion is present in only 6% of the general population [26]. Similarly, a polymorphic inversion has been reported at 15q11-q13 that gives rise to a *de novo* deletion resulting with the Angelman syndrome [27]. Two more striking examples are found in the Sotos syndrome [28] and the 17q21.31 microdeletion syndrome [8, 10, 29–31]. In each of these disorders, where a *de novo* microdeletion arises, every parent studied to date carries an inversion at the same region. All these inversions are enriched in segmental duplications at their breakpoints, leading to an increased susceptibility to non-allelic homologous recombination (NAHR), which in turn elevates risk for disease-causing rearrangements in the offspring. The typical presence of duplicated sequences at the inversion boundaries is also the major challenge for inversion detection.

Creation of a map of inversion polymorphisms will provide valuable information regarding their distribution and frequency in the human genome. Such a map will be important for future studies aimed to unravel how inversions and the segmental duplications architecture associated with inverted haplotypes contribute to genomic susceptibility to disease rearrangements. To fill this gap, the InvFEST [32] database aims to collect a comprehensive set of inversions reported in the literature. Currently InvFEST hosts 86 validated inversions, of which 14 are larger than 1 Mbp.

**Fig. 1:**
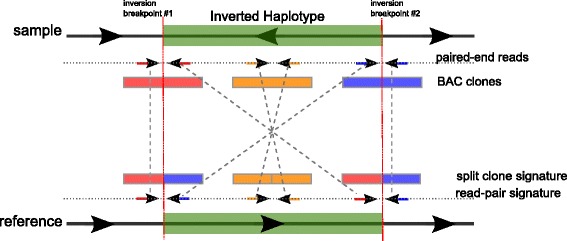
Sequence signatures used by the Valor algorithm. In the presence of an inverted haplotype in the sequenced genome, we look for both read pair and split clone signatures. Paired-end reads that span the inversion breakpoints will be mapped to the same strand with a large distance between them, instead of the concordant read pairs that map to opposing strands [1, 20]. Large insert clones will show mapping properties similar to the split read sequence signature [36], but since we do not have the full clone sequence, or sufficient coverage to assemble clones, we interrogate lengths of contiguous read mapping (Methods)

The common method to discover inversions is to analyze the read pair signature [1, 20], where the mapping strand of the read pairs spanning the inversion breakpoints will be different from what is expected (Fig. 1). For example, the Illumina platform generates read pairs from opposing strands but if the DNA frag ment spans an inversion breakpoint, the read pairs will have a discordant size and they will map to the same strand. When the inversion is large, the *real* mapping distance between pairs increases, therefore increasing the chance of incorrect mapping due to the common repeats that usually map at the inversion breakpoints and on other chromosomes. Another complication in accurate detection of large inversions arises as other types of SVs might occur within inversions or around inversion breakpoints further confusing the sequence signatures.

### Large molecule sequencing for long range contiguity

The HTS platforms generate data at very high rates with minimal cost. However, since both the HTS reads (100–150 bp for Illumina), and the DNA fragments are very short (350–500 bp), the mappability of the HTS data is dramatically reduced in repeat-rich regions that harbor most of the inversion breakpoints. Recently several methods have been developed to address these issues, and effectively to obtain information over a longer range. There are substantial differences between different approaches, yet, all have the same basic principles. First, the DNA is broken into very large molecules (10–100 Kb), then diluted and separated into a number of pools. Each pool is later barcoded and sequenced using the Illumina platform.

The first method that followed this procedure, called pooled clone sequencing (PCS), used fosmid and BACs to clone the input DNA [33] to generate 40-to- 50 Kb molecules. The alternative approaches avoid the cloning step and generate different average molecule sizes. These include the TruSeq Synthetic Long Reads (TSLR^1^), 10X Genomics linked- reads [34] (10XG), Dovetail Genomics (Chicago Method^2^), and CPT-Seq [35]. In the remainder of this paper, we collectively refer to these technologies as *long range sequencing* for simplicity.

Our approach to discover large genomic inversions up to tens of thousands base pairs using long range sequencing follows from the observation that, DNA molecules (sequenced as linked-reads or pooled clones) that span the inversion breakpoint will be split into two sections when mapped to the reference genome, also separated by a distance approximately the size of the inversion. We call this sequence signature as *split clones* (Fig. 1), which is similar to the split read sequence signature used by several SV discovery tools such as DELLY [21] and Pindel [36] but has the advantage that can span over repetitive regions. In contrast to split reads, incorrect mappings in large inversion regions using split clone signatures are less pronounced. This is because split clones are identified from many reads where each read pair is mapped concordantly, rather than using shorter alignments of single reads and then encompass larger regions that are longer than the repeats. Combining split-clone signatures with paired-end read signatures, we can distinguish the true paired-end read signatures and here even one pair of paired-end reads would suffice to detect the presence of the inversion and thus this approach allows us to detect very large inversions with low false discovery rate.

**Fig. 2:**
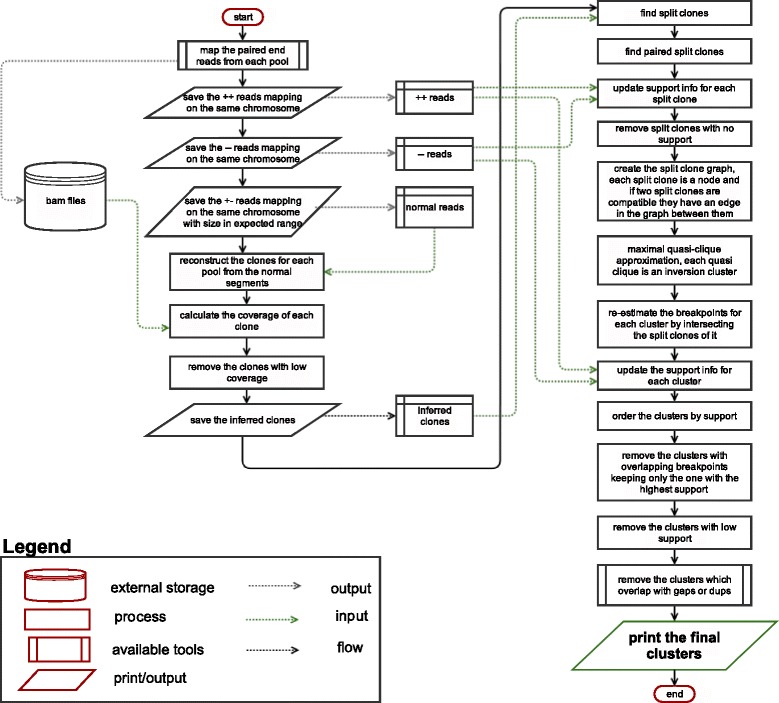
The Valor algorithm. We start with mapping paired-end reads for each pool. We then separate read pairs that map to the same strand, and generate two files for the two strands. We use the concordantly mapping reads to reconstruct clone locations, and calculate depth and breadth of coverage for the clones. Next, we remove clones with low coverage values, which results in the inferred clone locations. We identify the split clones from this list, and then find PSC, then we remove those with insufficient read pair support. Remaining PSCs are used to construct a graph, which we then use to find maximal quasi cliques that signal possible inversion locations. The clone and read pair support are then recalculated for the merged PSCs, those with low support and those that intersect with reference assembly gaps, or intersect with segmental duplications in both breakpoints are discarded

**Fig. 3:**
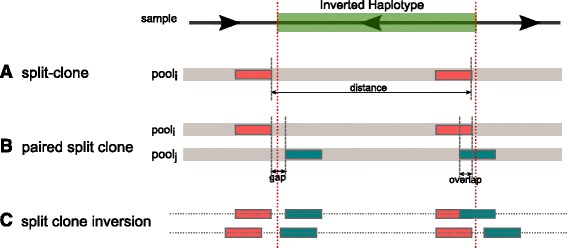
Clustering split clones to detect inversions. **a** We first identify clone locations that are shorter than the expected clone size, but when paired with another short clone found in the same pool, the total length sums up to a full clone length. We refer to such clones as “split clones”. **b** We then cluster pairs of split clones that are mapped to approximately the same breakpoints. Note that due to read mapping errors and our clone reconstruction heuristics, a split clone may be identified as spanning a breakpoint. **c** Finally we cluster multiples of split clones from different pools if they agree on breakpoint location and the size of the inversion. *gap*: size of the region between the start and end locations of split clones from different pools. *overlap*: size of the overlapping region of split clones from different pools

Based on these observations, we developed a novel combinatorial algorithm and statistical heuristics called Valor (**va**riation using **lo**ng **r**ange information) (Fig. 2). Briefly, Valor searches for both read pair and split clone sequence signatures (Fig. 3) using the mapping locations of long range sequencing reads, and requires split clones from different pools to cluster at the same putative inversion breakpoints (Methods). Ambiguity due to multiple possible pairings of split clones are resolved using an approximation algorithm for the maximal quasi clique problem [37]. Other tools such as VariationHunter [38] and CLEVER [39] use the Set-Cover or the equivalent maximum clique formulation [40] to cluster the variants. This approximation fails in clustering large inversions because it aims to detect complete cliques with low cardinality, which results in identifying a single breakpoint within repetitive regions of the genome (Additional file 1: 1.7).

Valor proves its potential when tested on simulated data, and it is able to discover previously characterized large inversions in the genome of a human individual (NA12878) using pooled BAC sequence data. Additionally we tested Valor using 10XG data generated from the same genome [34] and obtained similar results. Kitzman et al. [33] note that large clone (or molecule) size is required to span segmental duplication blocks, and smaller clones such as fosmids may not be sufficient to detect inversions around segmental duplications. In contrast we found on simulated data that fosmids perform as well as BAC clones, if not better, given that fosmids are still statistically larger than most segmental duplications. In conclusion, the theoretical minimum inversion size detectable by Valor is limited by clone length, i.e. 150 Kbp when BACs are used.

## Results

We designed various simulations to benchmark Valor in terms of precision and specificity under ideal conditions, and to compare performance of Valor with some of the popular SV discovery tools developed for whole genome shotgun (WGS) sequence data, such as LUMPY [22], DELLY [21], and GASVPro [25]. Additionally, we tested Valor using both PCS data [33] and 10X Genomics data generated from the genome of a human individual (NA12878) (Additional File 2).

### Simulations

Using VarSim [41], we implanted 686 simulated inversions in different sizes (500 bp to 10 Mbp) to the human reference genome (GRCh37). VarSim generated a diploid simulated genome, where 252/686 inversions intersected with another simulated inversion. VarSim uses databases of previously validated variation in the human genome and it allows for inserting few novel inversions (set to 1% by default). The simulated inversions are representative of realistic inversions. We then simulated a WGS library at 50X coverage using ART [42] to benchmark the WGS-based tools. The read length was 150 bp and the average fragment size was set to 600 bp with a standard deviation of 60 bp.

In order to test Valor, we randomly generated a set consisting of 300 pools of simulated fosmid (μ = 40 Kbp, σ = 10 Kbp) clones from the same simulated chromosomes at 5X physical coverage. We then simulated paired-end reads at 10X sequence coverage for each pool using ART with a read length of 150 bp, average fragment size of 600 bp and standard deviation of 60 bp. However, by random chance, no fosmid clones spanned breakpoints of 26 of 275 inversions larger than 40 Kbp, and 20 of 167 inversions greater than 80 Kbp.

We mapped both libraries to the reference genome using BWA-MEM [43], sorted and removed duplicates using SAMtools [44] and Picard [45], and realigned around indels using GATK IndelRealigner [46] with default parameters. We used Valor on the simulated clone data set, and three popular SV discovery tools (LUMPY, DELLY, and GASVPro) on the WGS simulation. In our tests, we required at least 50% reciprocal overlap between inversion intervals using the BEDtools suite [47]. Here, due to the presence of heterozygous inversions, a predicted inversion may intersect with two simulated inversions. We classify both such inversions as correctly identified in such cases.

We note that Valor can theoretically discover inversions at least as large as the clone size; however, using fosmid clones Valor was able to identify smaller inversions (<40 Kbp), although its performance and accuracy increase for larger variants (Additional file 1: 1.14). Of the 275 inversions (>40 Kbp), Valor was able to accurately detect 221 (80.4%) with only 7 false positives out of 198 calls (3.5%). The performance of Valor increases slightly for larger inversions (>80 Kbp): Valor discovered 139 out of 167 inversions (83.2%), with 7 false positive calls (5.3%). Among the WGS-based algorithms, DELLY performed the best in terms of recall (96.04% for inversions >40 Kbp and 94.81% for inversions >80 Kbp) but it suffered from a high false discovery rate (FDR, 40.9% and 53.48%). On the contrary, LUMPY had the best precision (100%) with low recall rates (67.44% and 60.9%). GASVPro performance was the lowest (Table 1); however, this is mainly due to the fact that GASVPro was designed for SVs smaller than 500 Kbp [25]. Overall, Valor showed the best balance of precision and recall rates.

**Table 1:** Simulation results on GRCh37

Tool	No. calls	TP	FP	FN	found	precision	recall
Inversions > 40 Kbp (*n* = 275)							
DELLY	780	461	319	19	256	59.10%	96.04%
LUMPY	174	174	0	84	191	100.00%	67.44%
GASVPro	475	9	166	266	9	1.89%	3.83%
Valor	198	191	7	54 (28)^a^	221	96.46%	77.96% (87.21%^b^)
Inversions > 80 Kbp (*n* = 167)							
DELLY	589	274	315	15	152	46.52%	94.81%
LUMPY	95	95	0	61	106	100.00%	60.90%
GASVPro	404	5	399	164	3	1.24%	2.96%
Valor	131	124	7	28 (8)^a^	139	94.66%	81.58% (93.94%^b^)

We implanted 686 inversions to the reference genome (GRCh37) using VarSim and simulated two libraries, one pooled fosmid clone sequencing library for Valor, and one WGS data set. 275 inversions had size >40 Kbp, and 167 were >80 Kbp. ^a^26 inversions (>40 Kbp) and 20 inversions (>80 Kbp) had no clone coverage. ^b^when inversions that had no clone coverage at breakpoints are removed. *TP* true positive, *FP* false positive, *FN* false negative. found: number of simulated inversions that intersect (>50% reciprocal) with calls. Precision: positive predictive value, calculated as TP/(TP + FP). Recall: sensitivity, calculated as TP/(TP + FN). Note that due to diploid simulated inversions, one call may intersect with multiple implanted inversions

We also performed three more simulation experiments to comprehensively test the Valor performance in the presence of other SVs and segmental duplications, and its ability to differentiate between inversions and inverted duplications (Additional file 1: 1.10).

### Characterizing false discovery using a haploid genome

All tools developed for SV discovery suffer from high false discovery rates. We used a haploid genome (CHM1) to characterize the false positive calls. To achieve this, we mapped Illumina WGS reads at 100X coverage to the CHM1.1 assembly [48]. Following the fact that the CHM1 cell line is haploid, and both the assembly and the WGS reads are derived from the same DNA resource, we do not expect to find any real SVs, but assembly errors may present themselves as variation. We applied the same analysis steps described above. Overall, DELLY predicted 5,578, GASVPro found 2,458, and LUMPY called 136 inversions, which shows the high false discovery rate of these tools.

Similarly, we simulated clones (average clone size 10 Kb, standard deviation 1 Kb) from the CHM1 assembly at 5X physical coverage. We then generated Illumina reads using ART at 10X sequence coverage per simulated clone. In this experiment, Valor returned no inversions as expected from the data.

### PCS data

We tested Valor using a real PCS data set generated from the genome of an individual of European descent (NA12878). We used genomic DNA from NA12878 to construct the library. High molecular weight DNA was isolated, partially EcoRI digested, and subcloned into pCC1BAC vector (Epicentre) to create a ~140 Kbp insert library using previously described protocols [49]. We then split a portion of this library to 3 sets of 96 pools each, with 230 clones per pool for set 1, 389 clones per pool for set 2 and 153 clones per pool for set 3. Each pool was expanded by direct liquid outgrowth after infection. We next constructed 96 barcoded sequencing libraries per each set, for a total of 288 sequencing libraries [50]. Libraries from each set were indexed with barcodes, combined and sequenced using the Illumina HiSeq platform (101 bp paired-end reads). Upon sequencing a total of 74,112 clones (22,080 in Set 1, 37,344 in Set 2 and 14,688 in Set 3) we obtained 3.38X expected physical depth of coverage. After read mapping and clone reconstruction, 87.58% of the genome was covered by one or more clones.

We mapped the paired-end reads from a total of 288 pools to the reference genome using BWA-MEM. Average fragment length of the paired-end reads was ~450 bp, with a standard deviation of ~98 bp. Using Valor, we reconstructed the clone locations, which showed an average clone length of ~140 Kbp and a standard deviation of ~40 Kbp. The mapping quality and coverage of data was very low and almost all the pools in set 3 were contaminated and did not map to any chromosome, leaving us with 2 sets (192 pools) and even lower coverage. Using Valor, we identified a total of 43 inversions larger than 200 Kbp (30 inversions >500 Kbp). We accurately detected all previously validated large inversions (>500 Kbp) (Table 2). Additionally, Valor was able to accurately predict a new inversion of 2 Mb in size at the 16p12.3 locus that we validated using fluorescent in situ hybridization (FISH).

**Table 2:** Summary of validation of inversions predicted in the genome of NA12878 using Valor

Chromosome	Coordinates	Result	InvFEST
chr8	6,922,489–12,573,597	confirmed [51, 52]	HsInv0501
chr15	30,823,312–32,859,062	confirmed [53]	HsInv1049
chr16	16,722,093–18,732,305	confirmed (this study)	HsInv0368
chr17	34,572,064–36,296,916	confirmed (InvFEST)	HsInv1048

Valor returns four coordinates for each inversion for two breakpoints. The coordinates above are the inner breakpoint predictions, and are from the GRCh37 reference genome. The InvFEST database reports inversions in NCBI reference build 36 coordinates, we thus converted the coordinates using the liftOver tool (https://genome.ucsc.edu/cgi-bin/hgLiftOver)

### 10X genomics linked reads

The linked-read sequencing (10XG) technology, developed recently by the 10X Genomics Incorporation sequencing technology, shows similarities to PCS, the DNA is sheared into large molecules that are pooled, barcoded, and sequenced using the Illumina platform. Reasoning from the similarities, we tested the Valor using 10XG data generated from the same (NA12878) genome [34], and two other individuals of the same trio (NA12877 and NA12882). This data set was provided as BAM files where approximately 480,000 pools were tagged with barcodes per individual and each pool included ~3 Mbp of sequence. Valor is not yet trio-aware, thus we analyzed each sample separately. As in the PCS test, Valor could detect all previously known large inversions (Table 3) and the same novel inversion that we validated using FISH (Table 3).

**Table 3:** Valor predictions in the CEPH trio genomes using 10X Genomics data

Chromosome	Coordinates	NA12877	NA12878	NA12882	Length (bp)	Notes
chr2	87,255,585–88,046,375	x	x		790,790	
chr2	110,616,644–111,256,595	x	x	x	639,951	
chr2	130,871,965–131,973,790		x		1,101,825	HsInv0669^(p)^
chr5	69,345,887–70,230,720	x	x	x	887,367	HsInv0690^(p)^
chr5	175,375,683–177,323,033		x		1,948,505	HsInv0273^(p)^
chr7	72,600,063–74,625,967	x	x		2,025,904	
chr7	149,700,848–153,805,583	x	x		4,104,827	HsInv0493^(p)^
chr8	8,004,167–12,382,355	x	x	x	4,379,883	HsInv0501, confirmed [51, 52]
chr9	38,936,292–40,159,168		x	x	1,222,876	
chr9	42,455,835–43,044,083		x		588,248	
chr15	22,680,455–28,680,554		x		6,000,099	HsInv0549, confirmed [53]
chr15	30,561,900–32,570,505		x	x	2,008,605	HsInv1049, confirmed^(v)^
chr16	15,342,375–16,594,696		x	x	1,252,321	HsInv0363^(p)^
chr16	16,696,143–18,748,024	x	x	x	2,051,881	HsInv0368, confirmed (this study)
chr16	21,771,066–22,591,511		x	x	820,445	
chr17	18,315,380–20,436,125	x	x		2,121,780	
chr17	28,978,332–30,399,013		x		1,420,681	
chr17	34,775,632–36,258,018		x	x	1,482,386	HsInv1048, confirmed^(v)^
chrY	6,238,807–9,635,568			x	3,400,156	confirmed [56]
chrY	25,590,628–28,369,119			x	2,781,421	

Inversions detected using 10XG. Check marks denote that the inversion is found in the genome of the corresponding individual. Those inversions marked with ^(p)^ are listed as predicted and those marked with ^(v)^ are listed as validated in InvFEST

### Whole genome sequencing of NA12878 and CHM1

The simulation results above showed that the Valor’s performance was comparable to the performance of LUMPY and DELLY, but benchmark on real data is warranted. We tested LUMPY, DELLY, and GASVPro using a PCR-free WGS data set at 50X coverage. We downloaded the BAM file that corresponds to NA12878 from the European Nucleotide Archive, sequenced as part of the “Illumina Platinum Genomes” project (ENA project ID: PRJEB3381), and applied the aforementioned SV discovery tools. DELLY was able to find all of the known large inversions; however, it returned a total of 3,094 inversion calls. We note that the known inversions that DELLY correctly identified had low quality score, thus simple score-based filtration from the large call set would also remove the true positives. LUMPY returned 161 inversion calls, but it failed to discover any of the previously known large inversions. GASVPro found 167 inversions of size >500 Kbp (48 after merging overlapping calls), and it was able to find one of the large inversions. Such results are expected since these tools are designed for smaller inversions.

In addition we used the CHM1 resource to test the sensitivity of WGS-based tools and compared the results from the WGS-based tools against a well-curated and validated database of SVs generated from the same library [23]. We mapped the CHM1 Illumina WGS reads to the human reference genome (GRCh37), and used the same tools to discover SVs. Briefly, out of 33 validated inversions reported in [23]), DELLY identified 13 and LUMPY found 8 inversions. GASVPro failed to characterized any known and validated inversions. Since there are no real PCS data sets generated from CHM1, and the average validated inversion length is ~7 Kb, we could not test Valor with the same approach.

### Experimental validation

**Fig. 4:**
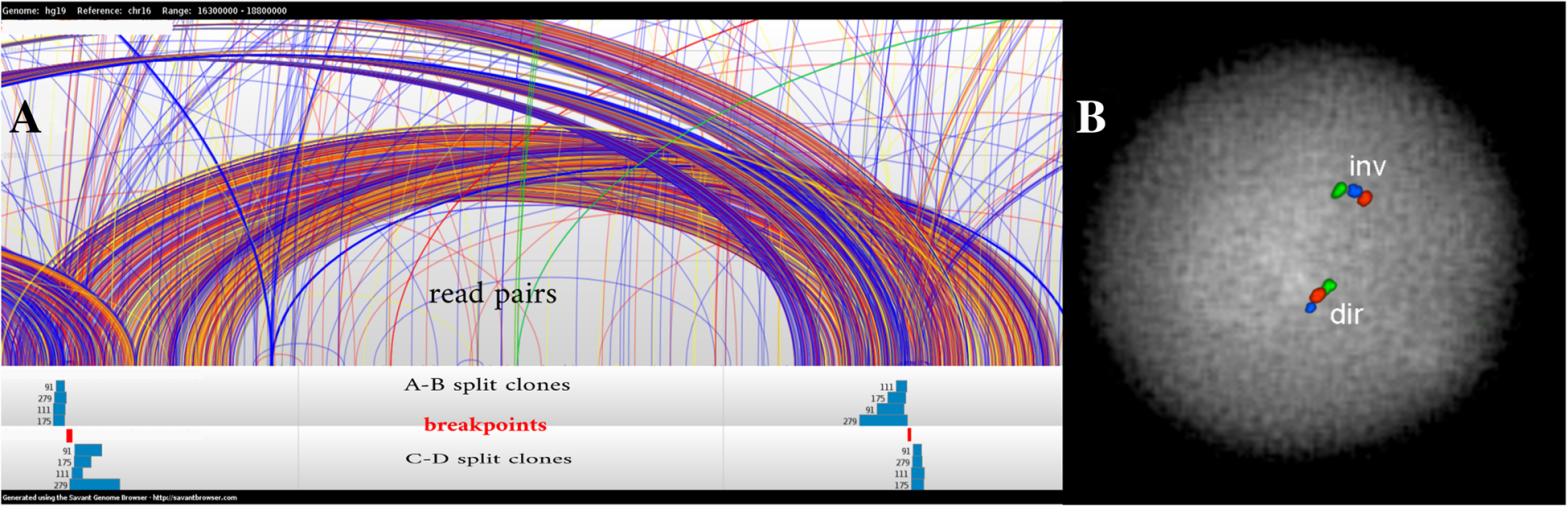
New inversion validated at chr16:16,722,093–18,732,305 (inner coordinates) in the NA12878 genome. **a** read-pair and split-clone signature used by Valor to detect the inversion. *a-b* split clones span the proximal breakpoint and *c-d* split clones span the distal. **b** experimental validation of the novel inversion discovered using interphase FISH (*green-red-blue*: direct, *green-blue-red*: inverted)

After the initial split clone clustering and maximal quasi clique approximation (Methods), we filtered those inversion clusters without sufficient read pair signature support (<2). Additionally, when PCS data is used, we also fi﻿ltered ﻿﻿those that were within segmental duplications or intersected with gaps. We found that Valor could correctly identify two inversions that were previously *validated* within the same genome (Table 2). These inversions include a 5 Mbp inversion in 8p23.1 [51, 52] and a 2 Mbp inversion in 15q13.3 [53]. We then selected 4 additional inversions for experimental validation that were not experimentally tested before (Table 2), but 3 of them could not be tested by FISH due to the amount of segmental duplications at the breakpoints. Our validation efforts resulted in the discovery of a novel, previously undocumented 2 Mb inversion at the 16p12.3 locus (Fig. 4). Moreover we compared our calls with previously validated inversions reported in InvFEST and found a match for one more inversion at the 17q12 locus.

## Discussion

In this paper, we presented a novel algorithm, Valor, to characterize large genomic inversions using two of the new sequencing methods that were initially developed to improve haplotype phasing. We showed that Valor is compatible with both PCS and linked-read sequencing technologies. Although it suffers from high FDR using real data (Table 2), Valor was able to identify all previously validated inversion events, and also discover a novel variant. Furthermore, Valor performed better with simulated data suggesting that the relatively lower performance with the NA12878 PCS data set may be improved with higher clone (or large molecule) coverage with more pools with higher sparsity (such as 10XG). We note that Valor still performed better than WGS-based tools in terms of large inversion detection sensitivity.

There are multiple directions that we can take to further improve Valor. First, to reduce the FDR, we can incorporate split read sequence signature [36], and we can perform local *de novo* assembly around the predicted breakpoint intervals with an approach similar to TIGRA [54]. However, since both of these methods need high and relatively uniform sequence coverage, they might not be suitable to directly apply to the data sets we used. Instead, it will be better to simultaneously use WGS data generated from the genome of the same individual. Since the PCS and 10XG methods also require additional WGS data for haplotype phasing, it can be expected to generate matching PCS/10XG-WGS data sets from the same genomes.

Another future research on Valor will be testing and improving its abilities to discover smaller, yet still large inversions (>10 Kbp). In this paper, we focused on inversions larger than 500 Kbp, because the upper size limit for GASVPro [25] algorithm is 500 Kbp, and only such large inversions can be reliably tested using FISH. Note that validating smaller inversions is a more difficult task using fiber FISH or PCR, if the breakpoints do not lie within unique regions. In addition, the clone size distribution should be tighter to ensure clone reconstruction method does not artificially “merge” split clones into a single interval. We still would like to investigate Valor’s performance using real fosmid data, but this may require additional algorithmic enhancements especially in the presence of nearby segmental duplications [33]. In this paper we present fosmid simulation experiments, and there is currently only one pooled fosmid sequencing dataset [33] generated from the genome of a Gujarati Indian individual (NA20847). However, this data set has even lower coverage and data quality, since this is the first data ever generated with PCS during its development phase. We would like to apply Valor to a newer fosmid-based dataset and evaluate its per formance with experimental validation. The Chicago method from Dovetail Genomics, the TruSeq Synthetic Long Reads [55], and the CPT-Seq technology [35] are other candidates for further Valor development.

Valor can also be extended to characterize other forms of large SV, including deletions, insertions, direct and inverted duplications. Each of these types of SVs present themselves with different split clone signatures (Additional file 1: 1.8). We also note that, determining the location of a segmental duplication event is yet a largely unsolved problem, even when long reads are used [23]. It may also be possible to discover translocations using split clones; however, chance of finding incorrect split clones will also increase, causing a reduction in the performance of maximal quasi clique approximation.

## Conclusions

Valor is the first algorithm that can discover large genomic inversions using HTS technologies. Our understanding of the phenotypic effects of inversions is still limited, and one of the reasons of this is the lack of reliable and cost effective methods to characterize such events. This is also true for other complex rearrangements such as duplications and translocations. Improvements in characterization of large complex rearrangements will help us better understand the biological mechanisms that lead to phenotypic difference, disease, and evolution.

## Methods

### Library preparation

We first generated a single whole-genome BAC library with long inserts (~140 Kbp). This procedure is a modification of the original haplotyping method previously described by Kitzman et al. (2011), that generates fosmid libraries with ~40 Kbp inserts. Here we used BAC clones, since long inserts are required to span the large duplication blocks where inversion breakpoints typically map [5, 33]. We then randomly partitioned the library into pools such that each pool is essentially a haploid mixture of clones derived from either the maternal or paternal DNA at each genomic location. High-throughput sequencing of each pool provides haplotype information for each clone in that pool. We mapped the paired-end reads generated for each pool separately to the human reference genome assembly using BWA-MEM [43]. We did not generate the 10XG data in this study, it was made freely available by the original authors [34] as pre-aligned BAM files.

### Inferring clones

We use only the concordantly mapped read pairs (i.e. fragment size within 3 standard deviations of the average) to infer the clone locations. We first merge spanning intervals of such pairs using BEDtools [47] merge command, while allowing up to a distance of 2 × μ_fragment_ between spanning intervals. Here we denote the spanning interval of a read pair as the interval between the starting map location of the proximal read and the end map location of the distal read. Depending on the data properties, the merge distance can be adapted to reconstruct the clones more precisely. For example when using 10XG data, due to very low clone coverage (~0.1X) the merge distance is set to larger values, up to 10 Kb. After the merge, we filter out those candidate clone intervals that are covered by less than 40% (i.e. *breadth* of coverage).

### Inversion discovery

After inferring clone locations, we also collect read pairs with inversion signature [1] (i.e. both reads mapping to the same strand). We then search for potential split clones within each pool by pairing inferred clone intervals where the summation of their lengths is within the expected size range for full clones. This is formulated as: μ_clone_ ± 3σ_clone_, where μ_clone_ is the mean clone size and σ_clone_ is the standard deviation.

Additionally we also require the distance between the split clones to be within the inversion size limits that we aim to discover (Fig. 3). Therefore, two regions r_k_ and r_l_ are predicted to be a split clone, denoted as SC_rk,rl_ if:

$$ \begin{array}{c}\kern2em {\upmu}_{\mathrm{clone}} - 3{\upsigma}_{\mathrm{clone}}\kern0.5em \le \kern0.75em \left|{\mathrm{r}}_{\mathrm{k}}\right| + \left|{\mathrm{r}}_{\mathrm{l}}\right|\kern0.75em \le {\upmu}_{\mathrm{clone}} + 3{\upsigma}_{\mathrm{clone}}\\ {} \min \_\mathrm{i}\mathrm{n}\mathrm{v}\_\mathrm{size}\kern0.5em \le \kern0.75em \left|{\mathrm{r}}_{\mathrm{k}}.\mathrm{start} - {\mathrm{r}}_{\mathrm{l}}.\mathrm{start}\right|\kern0.5em \le\ \max \_\mathrm{i}\mathrm{n}\mathrm{v}\_\mathrm{size}\end{array} $$

Assuming that the inferred clone locations are sorted by mapping locations, our algorithm can detect split clones in *O(n)* amortized run time, where *n* is the number of inferred clones. The constant coefficient increases with the sequence coverage.

We build inversion clusters by identifying two split clone pairs from different pools that are compatible (i.e. same breakpoint locations and inversion size). We denote such compatible pairs as a *pair of split clones* (PSC). Due to both mapping errors and biases caused by our sliding window approach we permit a gap or overlap between the split clones to be paired (Fig. 3b). We expect the inversion breakpoints to lie between these gaps. Two split clones SC_rk,rl_ and SC_rk’,rl’_ are compatible to be in the same PSC set, assuming r_k_/r_k’_ are located upstream of r_l_/r_l’_, if:

$$ \begin{array}{l}- \max\ \mathrm{overlap} < \left|{\mathrm{r}}_{\mathrm{k}'}.\mathrm{start} - {\mathrm{r}}_{\mathrm{k}}.\mathrm{start}\right| < \max\ \mathrm{gap}\\ {}- \max\ \mathrm{overlap} < \left|{\mathrm{r}}_{\mathrm{l}'}.\mathrm{start} - {\mathrm{r}}_{\mathrm{l}}.\mathrm{start}\right| < \max\ \mathrm{gap}\end{array} $$

Here we set the max gap = −1 × max overlap = μ_clone_. Note that adding more split clones to the same cluster will narrow down the gap size in breakpoint estimate. Not all of the split clones we identify signal an inversion event. In an ideal case, where there are no mapping errors, other forms of SV, or areas with low mappability may also show themselves as split clone signature for inversions. To ensure only split clones that signal a true inversion are detected, we also require read pair support for inversions [1, 20], and we discard any split clones that are not supported by read pairs. This step of the algorithm runs in *O(m + n)*, where *m* is the number of read pairs with inversion signature and *n* is the number of split clones.

Each pair of split clones gives a signature about the existence of an inverted haplotype. There may be many incorrectly identified split clone inversion signatures, or a short clone may have multiple potential “mate”s with similar properties. Therefore, clustering multiple split clone pairs that share inversion breakpoint locations and inversion lengths can help resolve the inversion breakpoints more accurately (Fig. 3c). To both resolve ambiguities from multiple possible split clone pairings, and unambiguously identify inversions, we construct an undirected graph, where each PSC is a node, and an edge between two nodes indicates that share predicted breakpoints.

We initially formulated the inversion detection using split clones as a Set-Cover problem similar to VariationHunter. We then observed in both simulation and real data sets that due to segmental duplications, deletions and nested inversions around the breakpoints, Set-Cover approximation selected only one of the inversion breakpoints correctly (Additional file 1: 1.7). We therefore formulate the problem as finding maximal quasi cliques in the inversion cluster graph.

A subgraph G’ = (V’, E’) of an undirected graph G = (V, E) is a (α, β)-Quasi Clique (0 < α, β < 1) if each node in V’ is connected to at least α.|V’| other nodes and |E’| > = β.|V’ |(|V’| − 1) edges where *n* = |G’| [37]. In other words, the ratios α and β represent how complete the subgraph G’ is. In contrast to the maximal clique problem or the set cover problem, this formulation allows for the existence of incomplete clusters, and tolerates some split clones to be included in a true cluster, and as a result, increases flexibility and avoids getting stuck in a local optimum.

We construct an inversion cluster graph G = (V, E) as follows. Each node in the graph denotes an inversion cluster, and each inversion will therefore represent a potential pair of inversion breakpoints. We put an edge between two nodes if the two representative in versions agree with breakpoint locations through simple intersection (they are compatible with each other). Formally,

$$ \begin{array}{c}\mathrm{V} = \left\{{\mathrm{v}}_i\Big|\kern0.35em {\mathrm{v}}_{\mathrm{i}}\kern0.1em \mathrm{denotes}\ \mathrm{a}\ \mathrm{P}\mathrm{S}\mathrm{C}\right\}\\ {}\mathrm{E} = \left\{\left({\mathrm{v}}_{\mathrm{m}}\kern0.1em ,\ {\mathrm{v}}_{\mathrm{n}}\right)\kern0.5em \Big|\kern0.5em \mathrm{breakpoints}\left({\mathrm{v}}_{\mathrm{m}}\right)\kern0.5em \cap \kern0.5em \mathrm{breakpoints}\left({\mathrm{v}}_{\mathrm{n}}\right)\right\}\end{array} $$

To find an approximate solution for the maximal quasi clique problem, we use an approximation algorithm previously suggested by [37], and we set the *tabu*, γ, and λ parameters to |graph|/10 *rounds*, 50%, and 60%, respectively. We obtained the values for these parameters by another grid optimization on experimental graphs depicting worst case scenarios (Additional file 1: 1.6).

When a quasi clique is found, the nodes within the clique denote a set of PSCs that are clustered together to mark an inversion. The breakpoint of this cluster is obtained by intersecting its split clones using a heuristic based on read pair support and the gap size. Next, the read pair support for the breakpoints within a distance is recalculated using the discordant read pairs. We report the final clusters after removing those that intersect with duplications and assembly gaps (>40%). A flowchart summarizing the Valor algorithm is available in Fig. 2.

### Experimental validation

We tested the presence of an inversion in the cell line of the NA12878 individual predicted to carry an inverted haplotype. For this purpose, we used interphase triple-color FISH using two probes inside and one outside the inversion.

## Additional files

Additional file 1: Supplementary Figures, Supplementary Tables, and additional text detailing methods and further benchmarking results. (PDF 2054 kb)
Additional file 2: Call sets. Call sets generated by Valor, DELLY, LUMPY, and GASVPro using both real data and simulations. (ZIP 72710 kb)

## Abbreviations

10XG: 10X genomics linked-reads
BAC: Bacterial artificial chromosomes
CNV: Copy number variation
FISH: Fluorescent in situ hybridization
HTS: High throughput sequencing
NAHR: Non-allelic homologous recombination
PCS: Pooled clone sequencing
PSC: Pair of split clones
SV: Structural variation
TSLR: TruSeq Synthetic Long Reads
Valor: Variation using long range information
WGS: Whole genome sequencing

## Footnotes

1
http://www.illumina.com/technology/next-generation-sequencing/long-read-sequencing-technology.html

2
http://dovetailgenomics.com/technology/
