# Supplementary material for: Discovery of large genomic inversions using long range information
Source: BMC Genomics. 2017 Jan 10;18:65. doi: 10.1186/s12864-016-3444-1 (PMC5223412; doi:10.1186/s12864-016-3444-1)
Supplement: Additional file 1: — Supplementary Figures, Supplementary Tables, and additional text detailing methods and further benchmarking results. (PDF 2054 kb) [file 12864_2016_3444_MOESM1_ESM.pdf]

Discovery of large genomic inversions using long range information

Marzieh Eslami Rasekh[1], Giorgia Chiatante[2], Mattia Miroballo[2], Joyce Tang[3], Mario Ventura[2], Chris T. Amemiya[3], Evan E. Eichler[4], Francesca Antonacci[2] and Can Alkan[1]

[1]Department of Computer Engineering, Bilkent University, 06800 Ankara, Turkey

[2]Department of Biology, University of Bari, Via Orabona 4, 70125 Bari, Italy

[3]Benaroya Research Institute, 1201 Ninth Avenue, 98101 Seattle, WA, United States

[4]Department of Genome Sciences and Howard Hughes Medical Institute, University of Washington, 3720 15th Avenue NE, 98195 Seattle, WA, United States

Contents

1 Supplementary notes 2
- 1.1 Probability of inversion discovery . . . 2
- 1.2 Variables and parameters . . . 3
- 1.3 Probability of clone overlap . . . 4
- 1.4 Clone reconstruction rate . . . 7
- 1.5 Parameter optimization of clone reconstruction . . . 7
- 1.6 Parameter optimization of the maximal quasi-clique . . . 7
- 1.7 The set cover approximation problem . . . 8
- 1.8 Other structural variation split clone signatures . . . 10
- 1.9 Correctness of VALOR on simulated data . . . 11
- 1.10 Robustness to other structural variations . . . 13
- 1.11 Comparison to other tools . . . 15
- 1.12 Robustness to segmental duplications . . . 15
- 1.13 VarSim simulation 1: testing prediction performance vs. inversion size. . . . 17
- 1.14 VarSim simulation 2: testing different parameters for WGS-based tools. . . . 18
- 1.15 Statistics on the real data of the NA12878 individual . . . 21
- 1.16 Inversions predicted on the real data of the NA12878 individual . . . 23
- 1.17 FISH validations . . . 25
- 1.18 Extra files . . . 26

1 Supplementary notes

1.1 Probability of inversion discovery

Assume there is an inversion with breakpoints B1 and B2. The probability of picking a clone of length clone.length uniformly from the genome of length genome.length such that it will pass one of the breakpoints with at least the distance of one paired-end read which is PE.length from the breakpoint is given as:

$$\mathrm{P1} = \mathrm{P}(\mathrm{clone.start} \in [\mathrm{B1} - \mathrm{clone.length} + \mathrm{PE.length}, \mathrm{B1} - \mathrm{PE.length}]) = \frac{\mathrm{clone.length} - 2\mathrm{PE.length}}{\mathrm{genome.length}} \tag{1}$$

Now we give the probability of having a clone of size clone.length which is ideally obtained by a Gaussian distribution with mean of clone.μ and standard deviation of clone.σ. The probability is approximated from the truncated normal distribution.

$$\begin{aligned}\mathrm{P2} = \mathrm{P}(\mathrm{clone}|\mathrm{clone.length}) &= \int_{x=\mathrm{clone.length}-1}^{\mathrm{clone.length}} \frac{\frac{1}{\mathrm{clone.}\sigma}\Phi\left(\frac{x-\mathrm{clone.}\mu}{\mathrm{clone.}\sigma}\right)}{\Phi\left(\frac{\mathrm{genome.length}-\mathrm{clone.}\mu}{\mathrm{clone.}\sigma}\right) - \Phi\left(\frac{-\mathrm{clone.}\mu}{\mathrm{clone.}\sigma}\right)} \\ &= \frac{\int_{x=\mathrm{clone.length}-1}^{\mathrm{clone.length}} e^{\frac{-(x-\mathrm{clone.}\mu)^2}{2}}}{\mathrm{clone.}\sigma\left(e^{\frac{-(\mathrm{genome.length}-\mathrm{clone.}\mu)^2}{2}} - e^{\frac{-\mathrm{clone.}\mu^2}{2}}\right)}\end{aligned} \tag{2}$$

Since S1 and S2 are independent, the probability of a clone passing B1 is:

$$\mathrm{P3} = \mathrm{P}(\mathrm{S1}|\mathrm{S2}) = \mathrm{P}(\mathrm{S1}) \times \mathrm{P}(\mathrm{S2}) \tag{3}$$

For all clones, we require at least one clone to cover B1, which means one occurrence of S3 in n times (Bernoulli). This probability is:

$$\mathrm{P4} = 1 - (1 - \mathrm{P3})^{\mathrm{n}} \tag{4}$$

where n is the number of clones and can be computed by:

$$\mathrm{n} = \frac{\mathrm{genome.length} \times \mathrm{physical.coverage}}{\mathrm{clone.}\mu} \tag{5}$$

Now we define P5 as the probability of having one clone covering B1 and another covering B2 when B2−B1+1 ≥ clone.length (given that B1 and B2 are far enough from each other) is:

$$\mathrm{P5} = \mathrm{P4}(\mathrm{n}) \times \mathrm{P4}(\mathrm{n}-1) = \left(1 - (1-\mathrm{P3})^{\mathrm{n}}\right).\left(1 - (1-\mathrm{P3})^{\mathrm{n}-1}\right) \tag{6}$$

Now we should calculate the probability of having two clones in the same pool. Assuming that the procedure of picking clones is independent from each other and the distribution is uniform:

$$\mathrm{P6} = \mathrm{P}(\mathrm{clone_i} \in \mathrm{pool_k} \ \& \ \mathrm{clone_j} \in \mathrm{pool_k}) = \frac{1}{\mathrm{pools.count}^2} \tag{7}$$

Finally we can define the probability of a **findable inversion** which means there is a clone passing B1 and another passing B2 while these two clones do not overlap:

$$\mathrm{P7} = \mathrm{P}(\mathrm{findable\ inversion}) = \mathrm{P5} \times \mathrm{P6} = \frac{\left(1 - (1-\mathrm{P3})^{\mathrm{n}}\right).\left(1 - (1-\mathrm{P3})^{\mathrm{n}-1}\right)}{\mathrm{pools.count}^2} \tag{8}$$

Here we have the probability of having clones such that a given inversion is findable.

1.2 Variables and parameters

In order to run VALOR, **the user only needs to specify the minimum and maximum inversion size to be detected**. All other parameters are automatically calculated based on the data set. Supplementary Table 1 explains the meaning and suggested value of each variable.

Supplementary Table 1: VALOR parameters

Paired-end read information		
**Parameter**	**Explanation**	**Value**
READ_LENGTH	Length of each read	*from data*
FRAG_MAX	Maximum fragment size from the paired-end reads in mapping	$\mu_{\text{fragment}} + 3\sigma_{\text{fragment}}$
FRAG_MIN	Minimum fragment size from the paired-end reads in mapping	$\mu_{\text{fragment}} - 3\sigma_{\text{fragment}}$
Clone reconstruction parameters[1]		
**Parameter**	**Explanation**	**Value**
WINDOW_SIZE	The minimum window size to look for potential clone seeds	μ_{fragment}
MIN_COVERAGE	The minimum coverage required for a window to be accepted as a clone seed	50-60%
EXTENSION	The distant from the edges of the clone seed to be extended to any fragment found, should be set to max fragment size	FRAG_MAX
Clone information for split clone discovery		
**Parameter**	**Explanation**	**Value**
CLONE_MEAN	The expected mean size of clones (i.e. 150 Kbp for BAC).	*from data*
CLONE_STD_DEV	The expected standard deviation of the clones.	*from data*
CLONE_MAX	The maximum possible clone length	$\mu_{\text{clone}} + 3\sigma_{\text{clone}}$
CLONE_MIN	The minimum possible clone length	$\mu_{\text{clone}} - 3\sigma_{\text{clone}}$
Inversion information		
**Parameter**	**Explanation**	**Value**
INV_MIN_SIZE	Minimum inversion size to find	*user specific*
INV_MAX_SIZE	Maximum inversion size to find	*user specific*
INV_GAP	The distance between two split clones, should allow for one normal clone size	μ_{clone}
INV_OVERLAP	The overlap allowed for split clones, should be set according to maximum fragment size for smaller inversions and to the size of a clone for> 500 Kbp	$-1\times$INV_GAP
INV_READ_LIMIT	The distance allowed around the split clones to find supporting reads, should allow for maximum fragment size	FRAG_MAX
Quasi-clique parameters[2]		
**Parameter**	**Explanation**	**Value**
QCLIQUE_LAMBDA	The minimum percentage of k-clique nodes which should be present in the subgraph to considered as a quasi-clique	0.5
QCLIQUE_GAMMA	The minimum percentage of k-clique edges which should be present in the subgraph to considered as a quasi-clique	0.6
QCLIQUE_TABU	Number of rounds a node can be removed and added to a quasi-clique	|InversionGraph|/10

[1] See optimized parameters in section 1.5. [2] See optimized parameters in section 1.6.

1.3 Probability of clone overlap

The probability of clones not overlapping in each pool is expected to be respectively low. However some inferred clones of size larger than expected were observed which we suspected them to be due to overlaps in some pools. The computational complexity of calculating the exact probability of overlap in a given set pool is too expensive ($O(n^{nm})$ where n is the number of clones and m is the length of the genome). In the real data of NA12878, there are approximately 230, 389, and 153 clones in each pool of set 1, 2, and 3, respectively. To evaluate the probability of clone overlap for each cutoff (number of clones overlapping), for maximum $2^{63} - 1$ test cases we extensively simulated a number of random clones in 288 pools (from normal distribution of μ=137 Kbp and σ=40 Kbp with cutoff 125 Kbp and 175 Kbp) and counted the average number of times there were x overlaps (for x=1 to total number of clones). Each test was stopped when the average number became stable to the thousands for 1000 consequent runs. This was repeated 1000 times and averaged for each cutoff (number of clones overlapping). The results are presented in the Supplementary Tables 2, 3, and 4 and Supplementary Figures 1,2, and 3. Figures were obtained by `RapidMiner`[1].

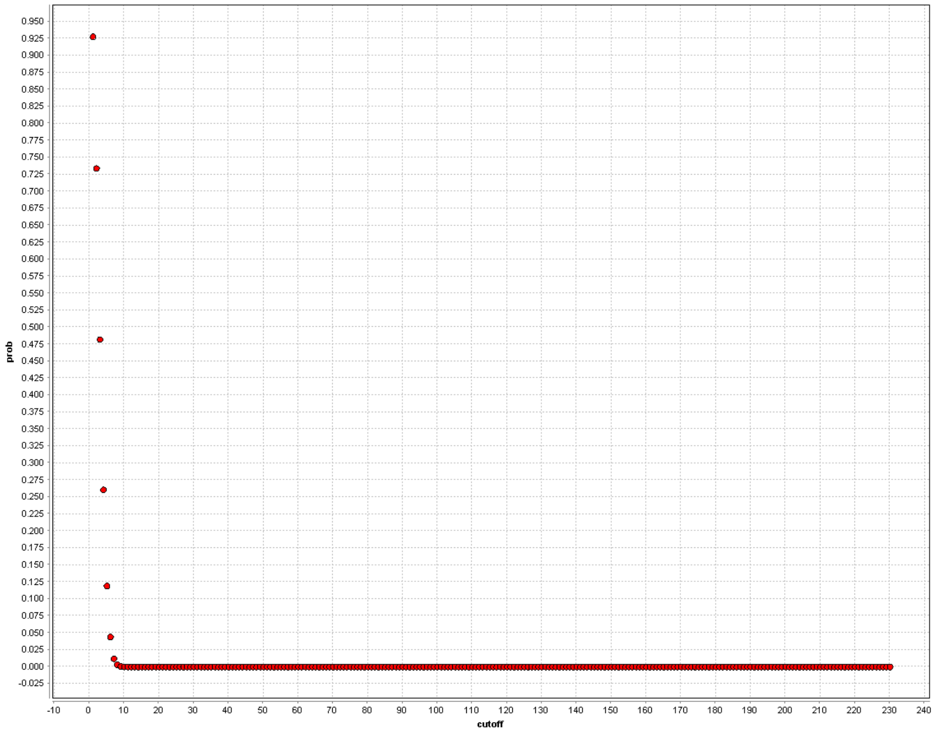

Supplementary Figure 1: Probability of overlapping for each number of clones estimated for set1 of pooled clone data of NA12878 with 230 clones per pool.

Supplementary Table 2: Exact values of overlapping probabilities estimated for set 1 of pooled clone data of NA12878 with 230 clones per pool.

cutoff	prob
1	92.789%
2	73.407%
3	48.212%
4	26.082%
5	11.916%
6	4.412%
7	1.210%
8	0.315%
9	0.072%
10	0.021%
11	0.009%
12	0.001%
13-230	0.00%

[1] https://rapidminer.com/

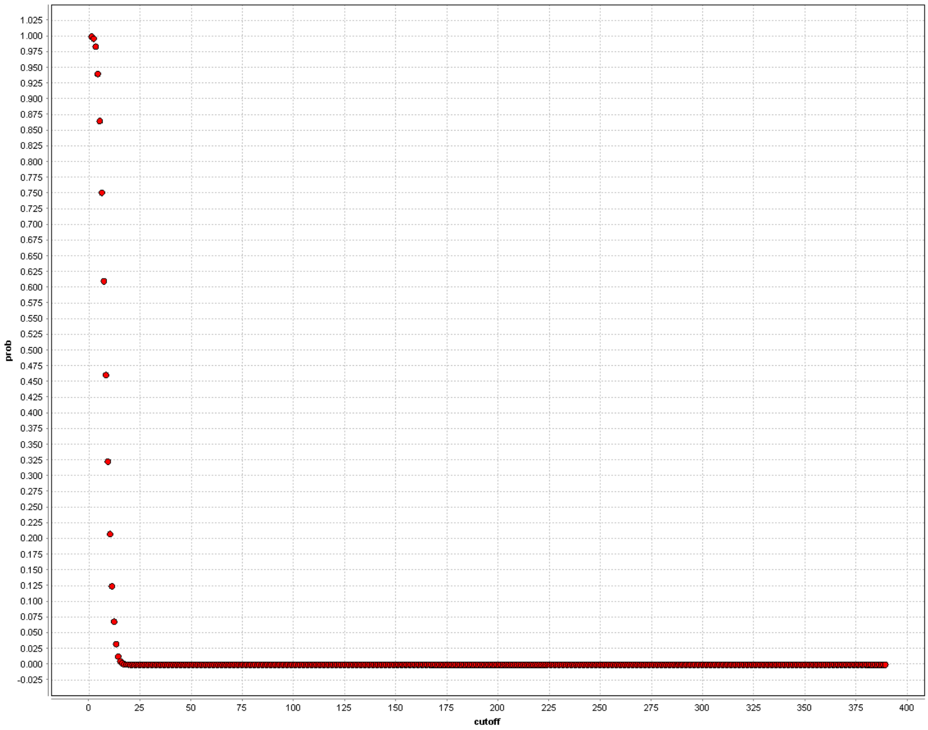

Supplementary Figure 2: Probability of overlapping for each number of clones estimated for set2 of pooled clone data of NA12878 with 389 clones per pool.

Supplementary Table 3: Exact values of of overlapping probabilities estimated for set2 of pooled clone data of NA12878 with 389 clones per pool.

cutoff	prob
1	99.967%
2	99.669%
3	98.380%
4	94.057%
5	86.551%
6	75.149%
7	61.036%
8	46.090%
9	32.326%
10	20.777%
11	12.479%
12	6.847%
13	3.280%
14	1.306%
15	0.549%
16	0.240%
17	0.075%
18	0.025%
19	0.011%
20	0.004%
21	0.001%
22	0.001%
23-389	0.00%

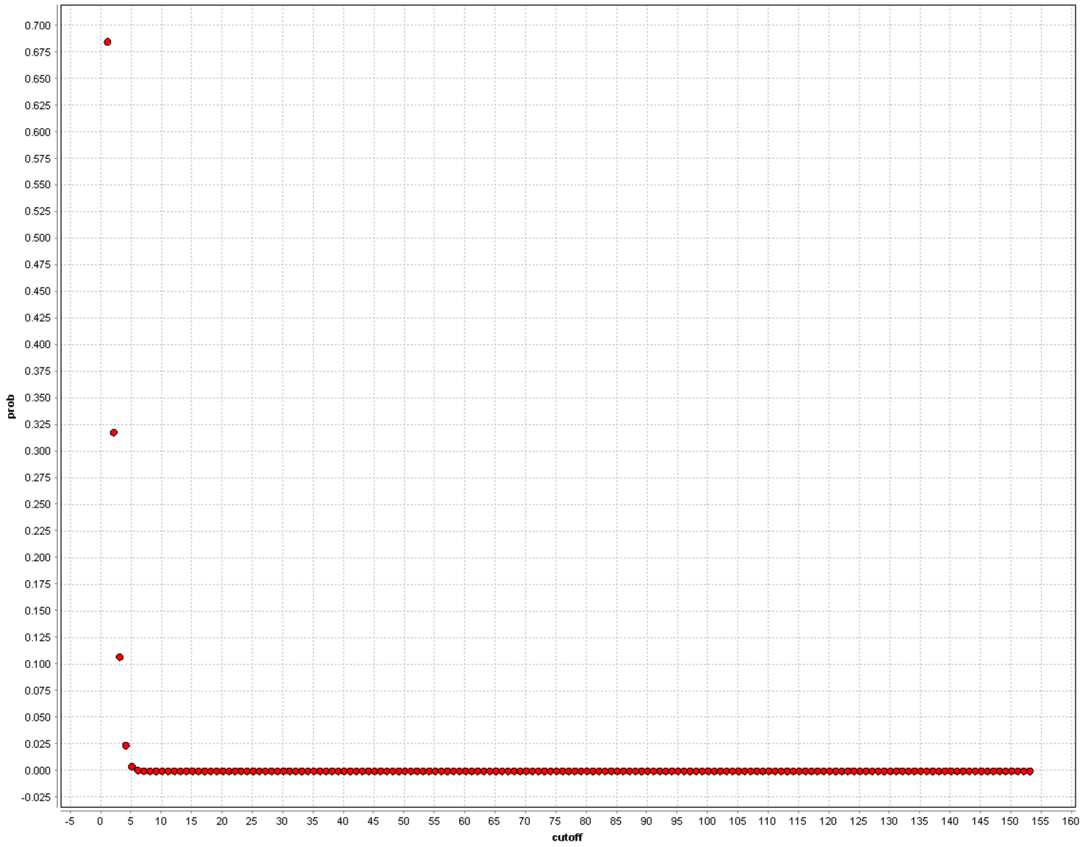

Supplementary Figure 3: Probability of overlapping for each number of clones estimated for set3 of pooled clone data of NA12878 with 153 clones per pool.

Supplementary Table 4: Exact values of of overlapping probabilities estimated for set3 of pooled clone data of NA12878 with 153 clones per pool.

cutoff	prob
1	68.498%
2	31.823%
3	10.719%
4	2.403%
5	0.436%
6	0.072%
7	0.013%
8	0.001%
9-153	0.00%

1.4 Clone reconstruction rate

In order to evaluate the number of clones correctly reconstructed, we simulated random clones on chromosome 1 with normally distributed sizes of (μ=150 Kbp , σ=40 Kbp) in 288 pools and generated random read pairs of size (μ=600 bp , σ=60 bp) with `wgsim`[2] at 3X, 5X, and 10X coverage and mapped them using BWA-MEM [1] and mrFAST [2] aligners. The number of clones that could be inferred correctly with $\geq$ 90% reciprocal intersection is given in the Supplementary Table 5. The parameters for the clone reconstruction were obtained by applying a grid optimization which is explained in section 1.5. The 10X, 15X and 20X coverage of mrFAST were not completed because of time restraints. As it can be observed VALOR relies on sufficient physical coverage (i.e. clones per pool) rather than sequencing coverage and can perform precisely in low sequence coverage.

Supplementary Table 5: Number of simulated clones correctly reconstructed by VALOR with at least 90% reciprocal intersection

	P	M	P/M	percentage
Total Clones	5,079	5,001	10,080	100.00%
Inferred by BWA at 3X read depth	4,480	4,313	8,793	87.23%
Inferred by BWA at 5X read depth	4,478	4,309	8,787	87.17%
Inferred by BWA at 10X read depth	4,478	4,310	8,788	87.18%
Inferred by BWA at 15X read depth	4,477	4,311	8,788	87.18%
Inferred by BWA at 20X read depth	4,477	4,307	8,784	87.14%
Inferred by mrFAST at 3X read depth	4,448	4,255	8,703	86.34%
Inferred by mrFAST at 5X read depth	4,452	4,264	8,716	86.47%

P and M are the the paternal and maternal DNA, respectively.

1.5 Parameter optimization of clone reconstruction

To reconstruct the clones from the normally mapping paired-end reads of each pool, we first look for windows of a minimum size which is covered by paired-end fragments by a pre-defined coverage rate. These well-covered windows are called clone seeds and are further extended to any existing fragment to the left or right at a given distance. In order to evaluate the best parameters for the minimum clone seed size, minimum coverage, and extension distance we applied a grid optimization on simulated data. Random clones on chromosome 1 with normally distributed sizes of (μ=150 Kbp , σ=40 Kbp) in 288 pools at 3X physical coverage were simulated and then fragmented with `wgsim` at 3X, 5X, 10X, 15X, and 20X coverage with size (μ=600 bp,σ=60 bp). The parameter grid used is given in the Supplementary Table 6. Due to duplicated regions and gaps and overlapping clones, not all clones can be precisely retrieved. It is worth mentioning that the reconstruction rate did not improve by increasing the coverage to 15X and 20X. Also, contrary to our expectation, mrFAST aligner could not perform as precisely as the BWA-MEM aligner. The optimum set of parameters were minimum clone seed length of 6.5 Kbp, minimum coverage of 50%, and extension distance of 1500 bp. However in the case of real data where split clones occur, the window size should be set to the maximum fragment size such to not miss any split clone smaller than the window size.

Supplementary Table 6: Grid for parameter optimization for clone reconstruction.

**parameter**	**min**	**max**	**step size**	**number of steps**
**min seed length**	3,000	146,000	500 up to 10,000<br>1,000 afterwards	160
**min coverage**	0.5	1.0	0.1	5
**read extension distance**	1,000	10,000	1,000	10
			**total**	8,000

1.6 Parameter optimization of the maximal quasi-clique

In order to find the optimum parameters for the maximal quasi-clique approximation algorithm proposed in [3], 100 random graphs each including 4 highly connected quasi-cliques were produced and on each, a grid optimization was applied. The graphs are not randomly expected cases but rather worse case scenarios that might occur and more similar to what we have observed in the real data set; meaning the neighboring nodes are connected with a higher probability than non adjacent nodes, there might exist many connections between the hidden quasi-cliques, and many edges within each quasi-clique might be missing. The algorithm used to optimize the parameters for the maximal quasi-clique approximation is given in Algorithm 1.

[2] https://github.com/lh3/wgsim

Algorithm 1 Quasi Clique Parameter Optimization

```
procedure MyProcedure
    for case ← 1 to 99
    G ← a new graph
    Sets[1..5] ← make 4 sets of nodes each of random size [4 × 2^⌊case/10⌋, 6 × 2^⌊case/10⌋]
    n ← |set1| + |set2| + |set3| + |set4|
    place all the nodes in G in order of the set and label them from 1 to n
    add another random [4 × 2^⌊case/10⌋, 6 × 2^⌊case/10⌋] nodes in between the nodes of G
    ∀i, j ∈ G.nodes add edge(i, j)
      with a probability of { 80%,                  if (node_i&nodej ∈ the same set)
                            { distance^-2 × 60%,    otherwise
      where distance is the difference of the order of the two nodes in the graph
    for each tabu ∈ {1, 2, ..., case/2} & lambda ∈ {0.1, 0.2, ..., 0.9} & gamma ∈ {0.1, 0.2, ..., 0.9}
      Solution ← MaximalQuasiClique(G, tabu, lambda, gamma)
      Score[case, n, tabu, lambda, gamma] =((number of real cliques) − (number of cliques found in Solution)
                    + (number of elements in each clique that were found)) / ⌊case/10⌋
    find the highest scoring point of (n, tabu, lambda, gamma)
```

* Note that no penalty is applied if the algorithm returns the set with additional nodes because this will not cause any difference in the final inversion detection.

It was observed that tabu ≤ 5 results into instability and slow convergence while values $\gg |nodes|/10$ result in poor performance. Thus, VALOR sets the tabu relative to the size of the nodes of the graph. Also, for small number of nodes (< 100) high lambda and gamma performed better, but as the number of nodes increased and the quasi-cliques overlapped more, increasing the lambda and gamma caused the algorithm to return only the largest quasi-clique with the most connected nodes of that clique. For larger graph sizes, lambda and gamma close to 0.5 performed better. Observing that the cliques in the first simulation data (physical coverage 3-4X) have hundred of nodes where lambda and gamma near 0.5 held the highest scores in that range, in the next phase, we ran the algorithm on simulation 1 data set (see section 1.9) on the inferred clones from BWA mapped read pairs with 10X coverage for a grid of lambda∈{0.4,0.5,0.6} and gamma∈{0.4,0.5,0.6}. As a result the optimum values for lambda and gamma were 0.5 and 0.6, respectively.

1.7 The set cover approximation problem

Initially we tried to formulate the split clone clustering problem as a set cover problem, similar to the approach used by Variation Hunter [4]. However in most cases we observe the set cover approximation returns inversion with one breakpoint precisely, while the other breakpoint is far from the exact locus. The problem is due to the nature of inversions where the breakpoints are located on duplications and highly repeated regions. For this reason, the inversion signatures, both split clones and read pairs, will have almost complete cliques for each inversion with many edges between the neighboring cliques. The equivalent for such a situation with a set cover formulation will be neighboring sets sharing the some elements as shown in Equation 9 and Supplementary Figure 4 :

$$\begin{aligned} U &= \{A, B, C, D, E, F, G, H, I, J\} \\ S &= \{\{A, B, C, D, H\}, \{C, D, E, F, H\}, \{F, G, H\}, \{H, I, J\}\} \end{aligned} \tag{9}$$

The set cover performing in any order will fail to recognize the most reliable breakpoint set because its greedy approach just chooses the set with the highest number of *new elements* which might lead to disfavoring other sets as their elements will become *found already*, and as a result, it will get stuck in a local optimum which is most likely the duplications near the breakpoint rather than the actual inversion itself. In contrast, if we choose a maximal quasi-clique approach, it can jump over these in-between-clique-edges and find the actual inversion. The effectiveness of the quasi-clique approach was observed on the second simulated data set (see section 1.10).The problem of set cover approximation can be solved to some extend by applying a semi-randomization technique in compare to ordered set cover approximation, but this will cause the approximation rate to be unpredictable, and therefore, unreliable. The SAVANT [5] visualization in Supplementary Figure 5 shows a real example of such in-between-clique-edges. Notice the humps made by the paired-end reads mapping around the HsInv1049 inversion of the NA12878 individual with breakpoint 1 at chr15:30,370,112−30,910,305 and breakpoint 2 at chr15:32,445,408−32,899,708. The quasi-cliques around the original inversion clique can be seen clearly in this picture.

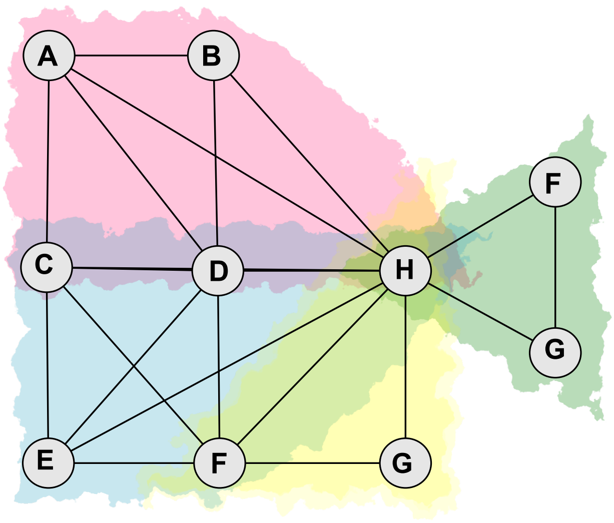

Supplementary Figure 4: Graph representation for the example given in (9). Each colored area represents a clique (equivalent to a set in the set cover formulation).

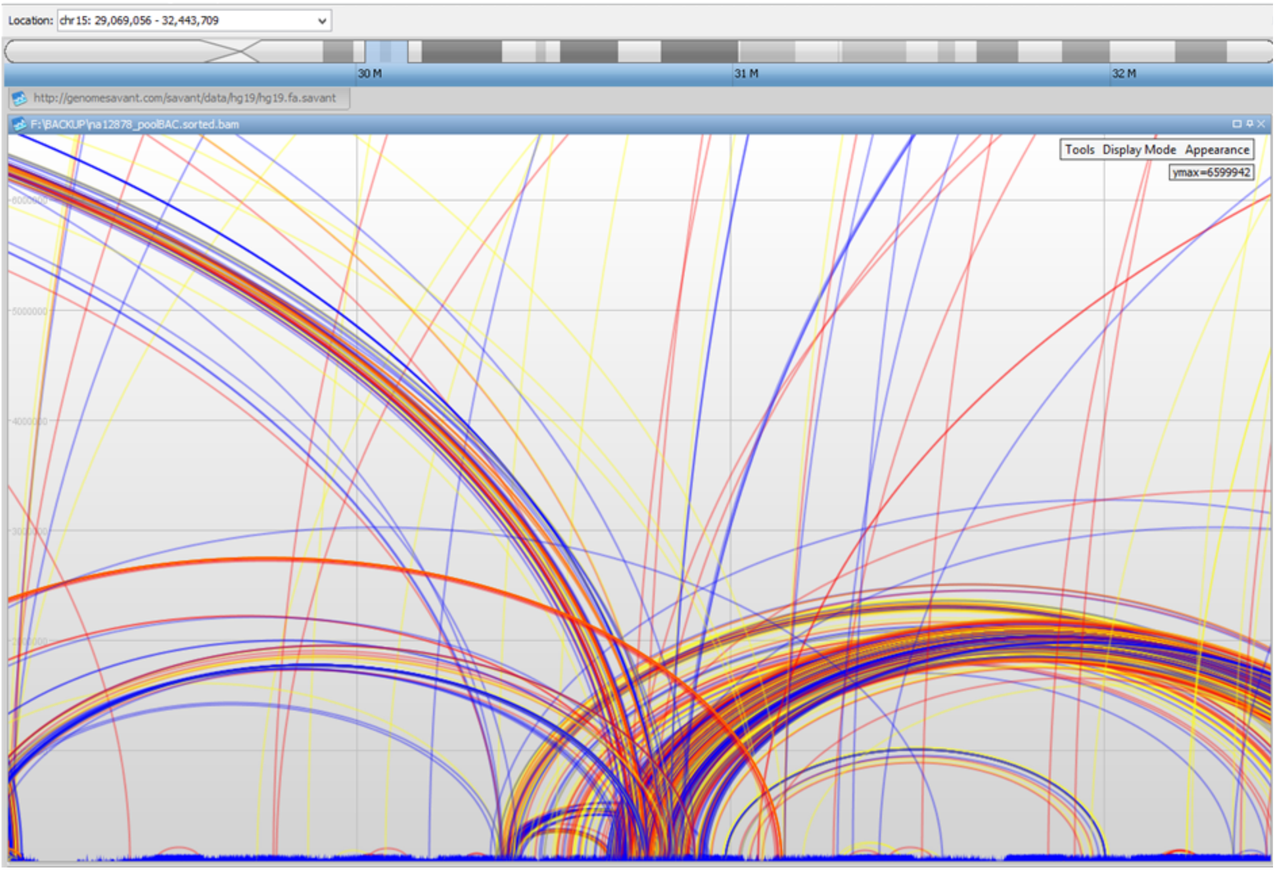

Supplementary Figure 5: Mapped paired-end reads around the HsInv1049 inversion of the NA12878 individual illustrated by SAVANT. Red arcs display the discordant length mapping paired-end reads, dark blue represent the one read inverted, yellow arcs represent the everted paired-end reads and the lighter blue forward, reverse, or concordant length paired-end reads. Reads were mapped by BWA in this example.

1.8 Other structural variation split clone signatures

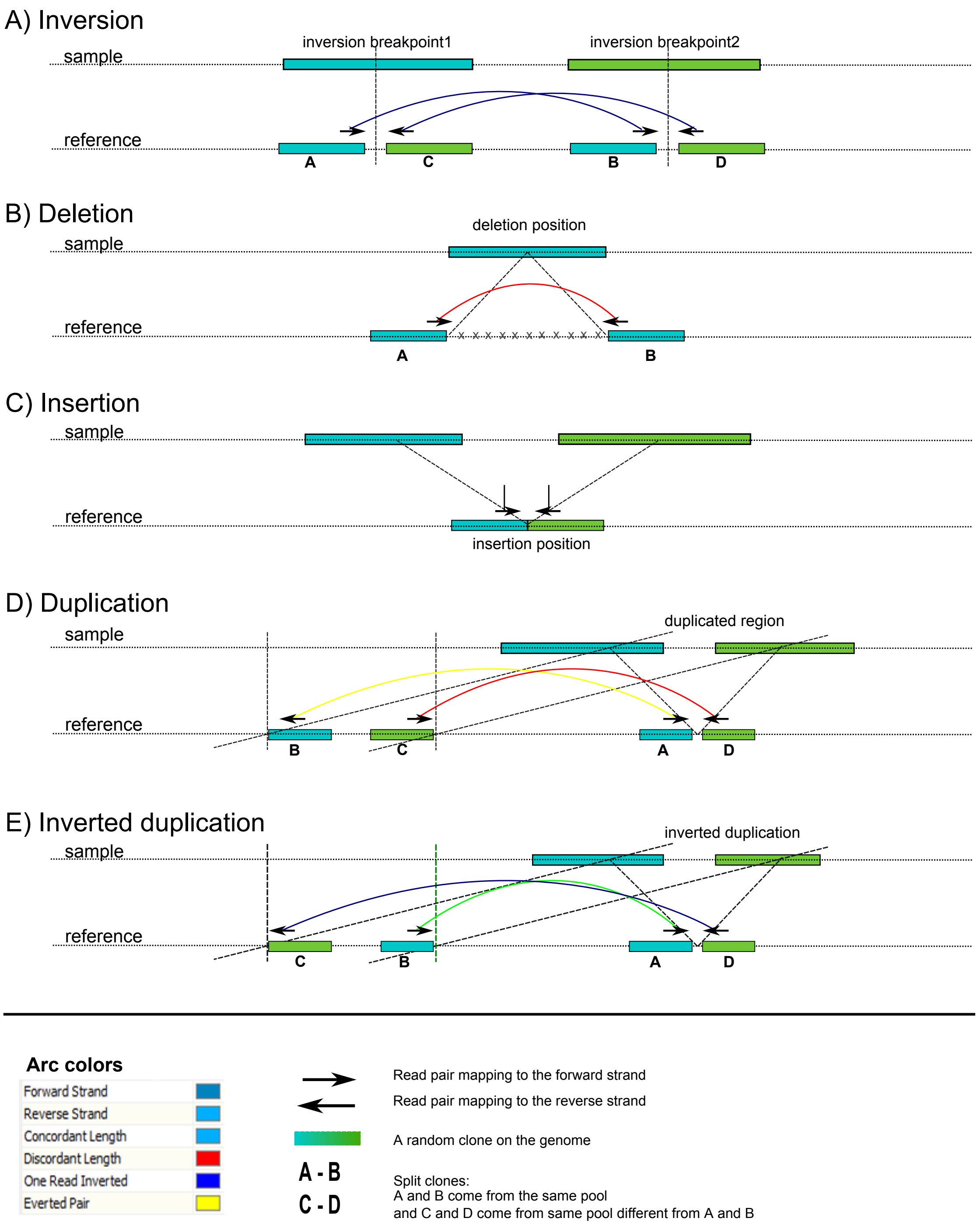

Supplementary Figure 6: Split clone and paired-read signature for different types of structural variations. Clones on the donor genome (sample) will break into a split clone on SV breakpoints and create specific read signatures as depicted.

1.9 Correctness of VALOR on simulated data

In order to evaluate the correctness of VALOR, 8 inversions of random size in range [500 Kbp , 10 Mbp] were implanted onto the human reference genome (GRCh37) chromosome 1 at random positions. The list of the inversions is given in Supplementary Table 7. Random clones with normal size distribution of (μ=150 Kbp and σ=4 Kbp) were simulated in 288 pools at ~3X physical coverage and read pairs with normally distributed size of (μ=600 bp and σ=60 bp) were generated by wgsim at 3X, 5X, and 10X coverage. The paired-end reads were mapped by BWA and mrFAST aligner and then, using VALOR, the inversions were called. Tables bellow show the results of each experiment.

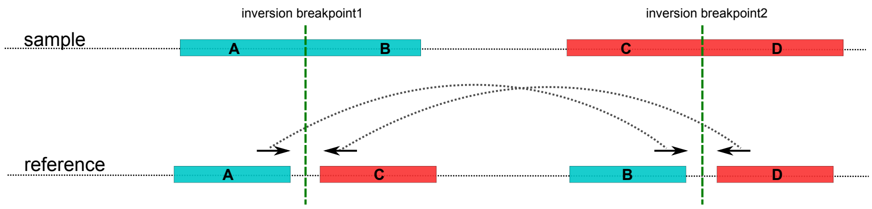

Supplementary Figure 7: Paired split clone signature of an inversion. Split clone AB from one pool and split clone CD from another pool will map accordingly to the reference genome with ++ reads supporting the AB split clone and −− reads supporting the CD split clone.

Supplementary Table 7: Inversions implanted on chromosome 1 for the first and second simulation experiments

ID	Start (bp)	End (bp)	Length (bp)	Genotype	SC_{1L}	SC_{1R}	$Detectable_1$	SC_{2L}	SC_{2R}	$Detectable_2$
Inv1	4,676,939	6,950,520	2,273,580	Het (P)	4	2	Y	0	3	N
Inv2	69,598,859	72,079,080	2,480,220	Het (M)	2	3	Y	10	6	Y
Inv3	76,232,699	82,398,900	6,166,200	Hom	7	6	Y	5+4	5+3	Y
Inv4	94,844,699	98,902,620	4,057,920	Hom	8	5	Y	3+4	5+2	Y
Inv5	107,694,119	109,006,800	1,312,680	Het (P)	1	4	Y	1	4	Y
Inv6	171,527,459	176,658000	5,130,540	Het (M)	2	7	Y	1	1	Y
Inv7	185,266,199	187,919,700	2,653,500	Hom	11	5	Y	2+3	3+2	Y
Inv8	190,600,559	198,012,420	7,411,860	Hom	6	7	Y	2+4	5+4	Y

SC_{1L} and SC_{1R} are the number of split clones on the left and right breakpoint of the first simulation, respectively and SC_{2L} and SC_{2R} are for the second simulation (complex rearrangements). The two split clone numbers for left/right breakpoints in the second simulation are separately shown for maternal and paternal homologs, as the deletions and duplications are simulated as heterozygous. $Detectable_1$ and $Detectable_2$ shows if there are sufficient number of split clones spanning the inversion breakpoint in the first and second simulation, respectively. Implanted inversions may be on one of the homologs (genotype=Het), or both (genotype=Hom). P: paternal, M: maternal copy.

Supplementary Table 8: Simulation 1 results at 3X sequence coverage using the ++/−− reads aligned by the BWA-MEM aligner

**chrom**	**left start**	**left end**	**right start**	**right end**	**AB**	**CD**	**PSC**	**Clu++**	**Clu−−**
chr1	4,676,834	4,676,941	6,950,341	6,950,523	4	10	9	4	10
chr1	69,598,666	69,598,985	72,078,771	72,079,641	11	7	24	11	7
chr1	76,232,635	76,232,701	82,398,750	82,398,912	8	13	24	8	13
chr1	94,844,639	94,844,699	98,902,086	98,902,652	5	14	27	5	14
chr1	107,694,087	107,694,177	109,006,650	109,006,857	1	4	6	1	4
chr1	171,527,266	171,527,459	176,657,976	176,658,043	11	9	20	11	9
chr1	185,266,111	185,266,201	187,919,391	187,920,258	4	11	21	4	11
chr1	190,600,382	190,600,561	198,012,231	198,012,420	10	11	24	10	11

++ is the read pair support on the AB split clones, −− is the read pair support on the CD split clone, PSC is the number of paired split clones in the cluster, Clu++ and Clu−− are the number of ++ and −− read pairs supporting the cluster.

Supplementary Table 9: Simulation 1 results at 5X sequence coverage using the ++/−− reads aligned by the BWA-MEM aligner

chrom	left start	left end	right start	right end	++	−−	PSC	Clu++	Clu−−
chr1	4,676,711	4,676,985	6,950,365	6,950,538	7	15	9	7	15
chr1	69,598,664	69,598,861	72,079,046	72,079,367	23	6	24	23	6
chr1	76,232,620	76,232,697	82,398,798	82,398,945	12	17	24	12	17
chr1	94,844,629	94,844,700	98,902,557	98,902,623	7	27	30	7	27
chr1	107,693,980	107,694,241	109,006,505	109,006,866	5	3	8	5	3
chr1	171,527,327	171,527,459	176,657,976	176,658,024	18	13	20	18	13
chr1	185,265,970	185,266,201	187,919,576	187,919,703	7	16	21	7	16
chr1	190,600,540	190,600,715	198,012,146	198,012,420	34	7	24	34	7

++ is the read pair support on the AB split clones, −− is the read pair support on the CD split clone, PSC is the number of paired split clones in the cluster, Clu++ and Clu−− are the number of ++ and −− read pairs supporting the cluster.

Supplementary Table 10: Simulation 1 results at 10X sequence coverage using the ++/−− reads aligned by the BWA-MEM aligner

chrom	left start	left end	right start	right end	++	−−	PSC	Clu++	Clu−−
chr1	4,676,780	4,676,941	6,950,466	6,950,521	24	28	9	24	28
chr1	69,598,822	69,598,861	72,078,996	72,079,083	50	21	24	50	21
chr1	76,232,586	76,232,701	82,398,805	82,398,903	43	50	24	42	50
chr1	94,844,576	94,844,700	98,902,553	98,902,623	19	67	30	19	67
chr1	107,694,058	107,694,121	109,006,701	109,006,835	16	7	8	16	7
chr1	171,527,415	171,527,459	176,657,931	176,658,002	31	32	20	31	32
chr1	185,266,045	185,266,200	187,919,633	187,919,702	15	46	21	15	46
chr1	190,600,465	190,600,561	198,012,259	198,012,420	53	18	24	53	18

++ is the read pair support on the AB split clones, −− is the read pair support on the CD split clone, PSC is the number of paired split clones in the cluster, Clu++ and Clu−− are the number of ++ and −− read pairs supporting the cluster.

Supplementary Table 11: Simulation 1 results at 3X sequence coverage using the alternative mappings given in the DIVET file obtained by the mrFAST aligner

chrom	left start	left end	right start	right end	++	−−	PSC	Clu
chr1	4,676,835	4,677,018	6,950,341	6,950,592	4	8	9	4
chr1	69,598,666	69,598,985	72,078,771	72,079,641	6	3	4	5
chr1	76,231,869	76,232,778	82,398,750	82,398,912	1	10	6	10
chr1	94,844,535	94,844,700	98,902,086	98,902,653	2	10	2	10
chr1	107,693,648	107,694,177	109,006,650	109,006,857	1	6	6	
chr1	171,527,266	171,527,531	176,657,911	176,658,039	5	4	5	4
chr1	185,266,111	185,266,215	187,919,391	187,919,926	3	5	3	5
chr1	190,600,382	190,600,608	198,012,231	198,013,032	5	5	2	5

++ is the inversion support on the AB split clones, −− is the inversion support on the CD split clone, PSC is the number of paired split clones in the cluster, Clu is the number of inversions supporting the cluster.

Supplementary Table 12: Simulation 1 results at 5X sequence coverage using the alternative mappings given in the DIVET file obtained by the mrFAST aligner

chrom	left start	left end	right start	right end	++	−−	PSC	Clu
chr1	4,676,711	4,676,985	6,950,365	6,950,538	4	8	9	4
chr1	69,598,664	69,598,883	72,078,822	72,079,367	14	3	14	3
chr1	76,232,554	76,232,694	82,398,798	82,399,020	6	13	6	13
chr1	94,844,346	94,844,710	98,902,404	98,902,651	5	17	5	17
chr1	107,693,980	107,694,241	109,006,586	109,006,866	9	2	9	2
chr1	171,527,327	171,527,502	176,657,816	176,658,127	9	5	9	5
chr1	185,265,970	185,266,210	187,919,576	187,919,739	4	11	4	11
chr1	190,600,257	190,600,715	198,012,146	198,012,435	6	5	6	5

++ is the inversion support on the AB split clones, −− is the inversion support on the CD split clone, PSC is the number of paired split clones in the cluster, Clu is the number of inversions supporting the cluster.

Supplementary Table 13: Simulation 1 results at 10X sequence coverage using the alternative mappings given in the DIVET file obtained by the mrFAST aligner

**chrom**	**left start**	**left end**	**right start**	**right end**	**++**	**−−**	**PSC**	**Clu**
chr1	4,676,780	4,676,942	6,950,411	6,950,537	13	11	9	11
chr1	69,598,738	69,598,858	72,078,993	72,079,090	15	13	15	13
chr1	76,232,586	76,232,693	82,398,805	82,398,960	16	28	16	28
chr1	94,844,576	94,844,696	98,902,473	98,902,620	18	46	16	46
chr1	107,694,009	107,694,135	109,006,701	109,006,808	20	3	8	3
chr1	171,527,353	171,527,461	176,657,868	176,658,081	17	25	20	25
chr1	185,266,045	185,266,192	187,919,389	187,919,743	6	29	6	29
chr1	190,600,465	190,600,557	198,012,259	198,012,496	34	16	24	16

++ is the inversion support on the AB split clones, −− is the inversion support on the CD split clone, PSC is the number of paired split clones in the cluster, Clu is the number of inversions supporting the cluster.

1.10 Robustness to other structural variations

In the second simulation other types of structural variations (SVs) were implanted near the same inversion breakpoints given in Supplementary Table 7 to observe the sensitivity of VALOR to the presence of the SVs around the breakpoints. The implanted SVs are given in Supplementary Table 14 and 15. Due to random cloning, the first inversion was not discoverable. All methods could retrieve the 7 discoverable inversions with no false positives except for mrFAST at 10X which suffered two false positive calls which shows that increasing the sequencing coverage too high will not always give better results. We have also shown that increasing the sequence coverage will worsen the clone reconstruction rate (see section 1.4).

Supplementary Table 14: Duplications implanted on chromosome 1 for the second simulation

No.	Target Locus(Mbp)	Genotype (target)	Source Locus (Mbp)	Genotype (source)	Length (Mbp)	Site	Type
1	77	Hom	75-77	Hom	2	Inv3	Direct
2	81	Hom	83-84	Hom	2	Inv3	Direct
3	95	Het (P)	92-94	Het (M)	2	Inv4	Direct
4	97	Hom	98-99	Het (M)	1	Inv4	Direct
5	109	Hom	106.5-107.5	Het (M)	1	Inv5	Direct
6	174	Het (M)	175-177	Het (M)	2	Inv6	Direct
7	200	Hom	Inv7.start-Inv7.end	Hom	3	-	Inverted
8	221	Het (M)	217.8-219	Het (M)	1.2	-	Inverted
9*	223	Het (P)	217.8-219	Het (P)	1.2	-	Inverted

Duplications 1-6 were in direct orientation, and 7-9 were inverted. Duplication #7 shares the same breakpoints with Inv7. *The duplication was inserted twice. Hom and Het are homozygous and heterozygous and P and M stand for paternal and maternal DNA.

Supplementary Table 15: Deletions implanted on chromosome 1 for the second simulation

No.	Locus (Mbp)	Length (Mbp)	Genotype	Site
1	4.5-4.67	0.17	Hom	Inv1
2	4.68-4.7	0.02	Hom	Inv1
3	6.5-6.9	0.4	Het (P)	Inv1
4	7.0-7.6	0.6	Het (P)	Inv1
5	65-69.5	4.5	Het (M)	Inv2
6	72-73	1	Het (P)	Inv2

Deletions are simulated as either heterozygous or homozygous (genotype, P: paternal, M: maternal copy for heterozygous simulations). Site: the ID of the closest implanted inversion (see Supplementary Table 7).

Supplementary Table 16: Simulation 2 results for BWA-MEM aligner at 3X sequence coverage

chrom	left start	left end	right start	right end	++	−−	PSC	Clu++	Clu−−
chr1	69,598,700	69,598,861	72,078,819	72,079,086	12	8	30	12	8
chr1	76,232,671	76,232,701	82,398,281	82,398,903	33	7	48	33	7
chr1	94,844,615	94,844,700	98,902,491	98,902,886	13	18	64	13	18
chr1	107,499,795	107,568,153	108,891,152	108,979,319	2	1	1	0	0
chr1	171,527,333	171,527,459	176,657,966	176,658,003	5	3	12	5	3
chr1	185,266,097	185,266,226	187,919,308	187,919,755	7	8	15	7	8
chr1	190,600,405	190,600,561	198,012,320	198,012,772	15	6	50	15	6

++ is the read pair support on the AB split clones, −− is the read pair support on the CD split clone, PSC is the number of paired split clones in the cluster, Clu++ and Clu−− are the number of ++ and −− read pairs supporting the cluster.

Supplementary Table 17: Simulation 2 results for BWA-MEM aligner at 5X sequence coverage

chrom	left start	left end	right start	right end	++	−−	PSC	Clu++	Clu−−
chr1	69,598,710	69,598,860	72,078,766	72,079,083	26	14	30	26	14
chr1	76,232,516	76,232,698	82,398,843	82,398,943	48	11	48	48	11
chr1	94,844,540	94,844,700	98,902,374	98,902,816	22	40	64	22	40
chr1	107,693,988	107,694,121	109,006,493	109,006,998	1	6	2	1	6
chr1	171,527,312	171,527,458	176,657,887	176,658,099	9	15	12	9	15
chr1	185,266,150	185,266,201	187,919,652	187,919,706	12	10	15	12	10
chr1	190,600,428	190,600,561	198,012,352	198,012,420	25	14	50	25	14

++ is the read pair support on the AB split clones, −− is the read pair support on the CD split clone, PSC is the number of paired split clones in the cluster, Clu++ and Clu−− are the number of ++ and −− read pairs supporting the cluster.

Supplementary Table 18: Simulation 2 results for BWA-MEM aligner at 10X sequence coverage

chrom	left start	left end	right start	right end	++	−−	PSC	Clu++	Clu−−
chr1	69,598,788	69,598,861	72,078,864	72,079,083	45	27	30	45	27
chr1	76,232,516	76,232,696	82,398,875	82,398,903	67	46	48	67	46
chr1	94,844,475	94,844,700	98,902,491	98,902,623	37	79	64	37	79
chr1	107,694,061	107,694,121	109,006,687	109,006,803	4	15	2	4	15
chr1	171,527,415	171,527,459	176,657,801	176,658,003	23	37	12	23	37
chr1	185,266,101	185,266,201	187,919,641	187,919,703	34	18	15	34	18
chr1	190,600,270	190,600,561	198,012,253	198,012,420	26	28	50	26	28

++ is the read pair support on the AB split clones, −− is the read pair support on the CD split clone, PSC is the number of paired split clones in the cluster, Clu++ and Clu−− are the number of ++ and −− read pairs supporting the cluster.

Supplementary Table 19: Simulation 2 results for mrFAST aligner at 3X sequence coverage using alternative mappings given in the DIVET file

chrom	left start	left end	right start	right end	++	−−	PSC	Clu
chr1	69,598,538	69,598,883	72,078,971	72,079,224	4	2	4	2
chr1	76,232,548	76,232,843	82,398,281	82,399,081	13	3	13	3
chr1	94,844,416	94,844,701	98,902,491	98,902,768	7	10	6	10
chr1	107,693,958	107,694,370	109,006,676	109,006,917	1	4	1	4
chr1	171,527,333	171,527,501	176,657,804	176,658,190	4	3	4	3
chr1	185,266,097	185,266,226	187,919,308	187,919,755	4	4	4	4
chr1	190,600,405	190,600,565	198,012,320	198,012,473	7	4	7	4

++ is the inversion support on the AB split clones, −− is the inversion support on the CD split clone, PSC is the number of paired split clones in the cluster, Clu is the number of inversions supporting the cluster.

Supplementary Table 20: Simulation 2 results for mrFAST aligner at 5X sequence coverage using alternative mappings given in the DIVET file

chrom	left start	left end	right start	right end	++	−−	PSC	Clu
chr1	69,598,710	69,598,861	72,078,767	72,079,099	10	10	10	10
chr1	76,232,516	76,232,698	82,398,623	82,398,943	22	6	22	6
chr1	94,844,540	94,844,733	98,902,374	98,902,620	12	28	12	28
chr1	107,693,989	107,694,214	109,006,493	109,006,998	1	6	1	6
chr1	171,527,312	171,527,459	176,657,887	176,658,100	7	7	7	7
chr1	185,266,067	185,266,195	187,919,478	187,919,793	4	5	4	5
chr1	190,600,428	190,600,557	198,012,265	198,012,598	9	7	9	7

++ is the inversion support on the AB split clones, −− is the inversion support on the CD split clone, PSC is the number of paired split clones in the cluster, Clu is the number of inversions supporting the cluster.

Supplementary Table 21: Simulation 2 results for mrFAST aligner and 10X coverage using alternative mappings given in the DIVET file.

chrom	left start	left end	right start	right end	++	−−	PSC	Clu
chr1	69,598,738	69,598,865	72,078,911	72,079,129	13	17	12	17
chr1	76,232,589	76,232,695	82,398,757	82,398,924	12	21	12	21
chr1	94,844,589	94,844,705	98,902,486	98,902,657	14	14	14	14
chr1	107,694,017	107,694,118	109,006,506	109,006,846	7	1	2	1
chr1	145333689	145342656	148329447	148321572	2	1	9	0
chr1	145333653	145342742	148015682	148011520	2	7	1	1
chr1	171,527,320	171,527,463	176,657,906	176,658,084	9	7	10	7
chr1	185,266,027	185,266,199	187,919,523	187,919,764	6	26	49	5
chr1	190,600,446	190,600,557	198,012,281	198,012,420	26	7	14	7

++ is the inversion support on the AB split clones, −− is the inversion support on the CD split clone, PSC is the number of paired split clones in the cluster, Clu is the number of inversions supporting the cluster.

1.11 Comparison to other tools

In order to compare our algorithm with other tools available in the literature we ran LUMPY [6], INVY [7] (DELLY2), and VariationHunter [4] on paired-end reads of chromosome 1 with the implanted inversions given in Supplementary Table 7 and the extra SVs (simulation 2 data set) at 3X, 5X, and 10X sequence coverage. LUMPY and INVY failed to find any inversions. VariationHunter could find only one inversion. The deletions it found are all incorrect.

Supplementary Table 22: Results from Variation Hunter on the simulation 2 data. At each coverage the same result was obtained.

Chr:chr1 Start Outer:4,679,679 Start Inner:4,679,924 End Inner:4,700,045 End Outer:4,700,354<br>SVtype:D sup:3 Sum Weight:0 AvgEditDits:6.333333<br>LibSup:3 LibHurScore:3 AvgEditDistInd:6.33333 minDelLen:19,531 maxDelLen:20,331
Chr:chr1 Start Outer:4,727,092 Start Inner:4,727,354 End Inner:5,127,561 End Outer:5,127,787<br>SVtype:D sup:6 Sum Weight:0 AvgEditDits:2.666667<br>LibSup:6 LibHurScore:6 AvgEditDistInd:2.66667 minDelLen:399,504 maxDelLen:400,304
Chr:chr1 Start Outer:6,927,112 Start Inner:6,927,358 End Inner:6,947,467 End Outer:6,947,767<br>SVtype:D sup:4 Sum Weight:0 AvgEditDits:4.500000<br>LibSup:4 LibHurScore:4 AvgEditDistInd:4.5 minDelLen:19,555 maxDelLen:20355
Chr:chr1 Start Outer:107,693,862 Start Inner:107,694,464 End Inner:109,006,483 End Outer:109,006,950<br>SVtype:V sup:5 Sum Weight:0 AvgEditDits:2.400000<br>LibSup:5 LibHurScore:5 AvgEditDistInd:2.4

1.12 Robustness to segmental duplications

In order to show the robustness of the VALOR algorithm to segmental duplications (SDs), in another simulation, 4 inversions with breakpoints spanning on SDs were places on chromosome 22 of the human genome (GRCh37). The implanted inversions are given in the Supplementary Table 23.

Supplementary Table 23: Inversions implanted on chromosome 22 with breakpoints placed on structural duplications

chromosome	start locus	end locus	heterozygous or homozygous
chr22	18,999,999	20,145,000	heterozygous (paternal)
chr22	22,606,699	29,075,000	homozygous
chr22	33,999,999	36,524,000	homozygous
chr22	42,105,089	44,963,000	heterozygous (maternal)

Then random BAC (μ=150 Kbp , σ=40 Kbp, cutoff=100 Kbp, clones per pool = 5) and random fosmids (μ=40 Kbp , σ=10 Kpb, cutoff=30 Kbp, clones per pool=16) clones were simulated in 288 pools ($\sim$4X physical coverage) and fragmented by wgsim with 3X, 5X and 10X coverage. Average fragment size was 600 bp with standard deviation of 60 bp. The paired-end reads were mapped with the BWA-MEM aligner. The results are given in the following tables.

Supplementary Table 24: Simulation 3 results for BAC clones mapped with the BWA-MEM aligner at 3X sequence coverage

chrom	left start	left end	right start	right end	++	−−	PSC	Clu ++	Clu−−
chr22	18,999,825	18,999,998	20,145,001	20,145,358	3	6	9	5	6
chr22	22,606,790	22,607,089	29074917	29,075,100	15	6	22	20	6
chr22	33,999,534	34,000,000	36,523,854	36,524,146	1	10	20	13	10
chr22	42,105,031	42,105,090	44,962,358	44,963,003	7	4	9	7	4

++ is the inversion support on the AB split clones, −− is the inversion support on the CD split clone, PSC is the number of paired split clones in the cluster, Clu++ and Clu−− are the number of inversions supporting the cluster.

Supplementary Table 25: Simulation 3 results for BAC clones mapped with the BWA-MEM aligner at 5X sequence coverage

chrom	left start	left end	right start	right end	++	−−	PSC	Clu ++	Clu−−
chr22	18,999,747	19,000,000	20144833	20,145,367	8	4	9	10	4
chr22	22,606,888	22,607,068	29074930	29,075,002	23	12	22	23	12
chr22	33,999,937	34,000,000	36,523,984	36,524,017	15	19	20	15	19
chr22	42,104,773	42,105,090	44,963,001	44,963,112	7	7	9	10	4

++ is the inversion support on the AB split clones, −− is the inversion support on the CD split clone, PSC is the number of paired split clones in the cluster, Clu++ and Clu−− are the number of inversions supporting the cluster.

Supplementary Table 26: Simulation 3 results for BAC clones mapped with the BWA-MEM aligner at 10X sequence coverage

chrom	left start	left end	right start	right end	++	−−	PSC	Clu ++	Clu−−
chr22	19,000,000	19,000,000	20,145,002	20,145,002	15	8	9	15	8
chr22	22,606,979	22,607,000	29,075,003	29,075,001	43	17	22	43	17
chr22	33,999,971	34,000,028	36,523,951	36,524,002	30	26	20	30	26
chr22	42,105,090	42,105,140	44,963,001	44,963,002	24	17	9	15	8

++ is the inversion support on the AB split clones, −− is the inversion support on the CD split clone, PSC is the number of paired split clones in the cluster, Clu++ and Clu−− are the number of inversions supporting the cluster.

Supplementary Table 27: Simulation 3 results for FOSMID clones mapped with the BWA-MEM aligner at 3X sequence coverage

chrom	left start	left end	right start	right end	++	−−	PSC	Clu ++	Clu−−
chr22	18,999,962	19,000,004	20144782	20,145,002	6	3	6	6	3
chr22	22,606,369	22,607,000	29074592	29,075,584	1	11	20	6	11
chr22	33,999,762	34,000,000	36,523,799	36,524,002	8	7	12	10	7
chr22	42,105,006	42,105,246	44,962,974	44,963,094	2	6	1	2	6

++ is the inversion support on the AB split clones, −− is the inversion support on the CD split clone, PSC is the number of paired split clones in the cluster, Clu++ and Clu−− are the number of inversions supporting the cluster.

Supplementary Table 28: Simulation 3 results for FOSMID clones mapped with the BWA-MEM aligner at 5X sequence coverage

chrom	left start	left end	right start	right end	++	−−	PSC	Clu ++	Clu−−
chr22	18,999,775	19,000,000	20,145,001	20,145,002	6	6	6	6	6
chr22	22,607,000	22,607,000	29,075,003	29,075,093	17	15	15	17	15
chr22	33,999,946	34,000,000	36,523,649	36,524,024	15	18	20	15	18
chr22	42,105,090	42,105,308	44,963,001	44,963,004	3	9	2	3	11

++ is the inversion support on the AB split clones, −− is the inversion support on the CD split clone, PSC is the number of paired split clones in the cluster, Clu++ and Clu−− are the number of inversions supporting the cluster.

Supplementary Table 29: Simulation 3 results for FOSMID clones mapped with the BWA-MEM aligner at 10X sequence coverage

chrom	left start	left end	right start	right end	++	−−	PSC	Clu ++	Clu−−
chr22	18,999,910	19,000,000	20,145,001	20,145,002	15	19	6	15	19
chr22	22,607,000	22,606,998	29,075,003	29,075,004	38	21	20	38	21
chr22	33,999,954	34,000,000	36,524,001	36,524,002	23	22	20	23	22
chr22	42,105,009	42,105,090	44,962,824	44,963,002	13	19	2	13	19

++ is the inversion support on the AB split clones, −− is the inversion support on the CD split clone, PSC is the number of paired split clones in the cluster, Clu++ and Clu−− are the number of inversions supporting the cluster.

1.13 VarSim simulation 1: testing prediction performance vs. inversion size.

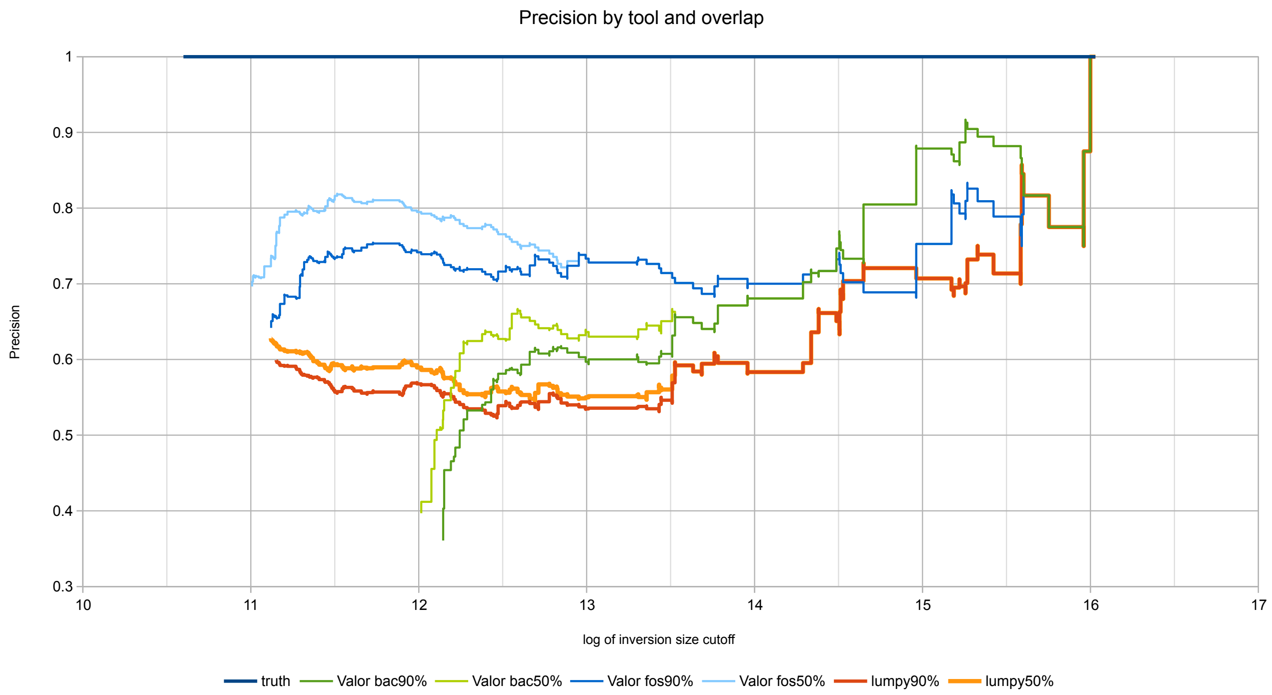

Supplementary Figure 8: Performance of VALOR increases for larger inversions.

1.14 VarSim simulation 2: testing different parameters for WGS-based tools.

We performed a second simulation using VarSim to investigate the effect of different parameters on WGS tools and compare against VALOR. We used VarSim with default parameters and inserted a total 616 inversions to the GRCh37 reference genome. 270 inversions had size larger than 40Kbp and 160 had size larger than 80Kbp.

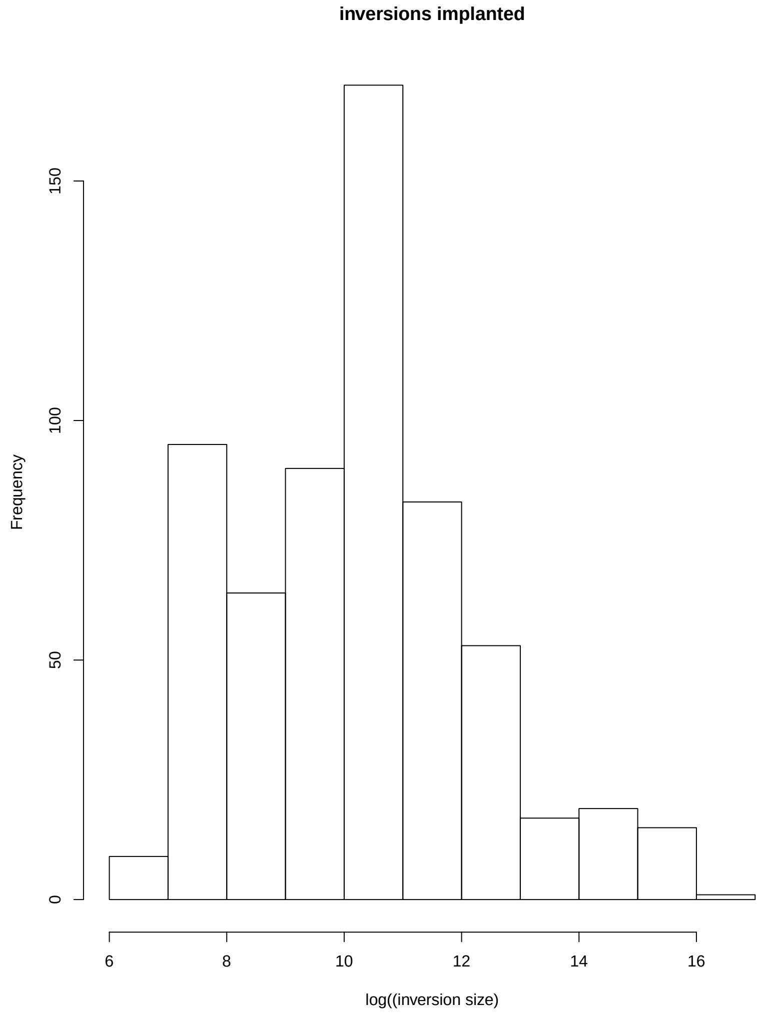

Supplementary Figure 9: Histogram of log of inversion sizes implanted on the GRCh37 male genome with VarSim

For the WGS tools, DELLY2 and LUMPY the genome sequence was simulated at 60X coverage using ART Illumina paired end read simulator with default parameters. We used DELLY2 and LUMPY with the default parameters. We did not test GASVPro due to its poor performance in the previous simulation as presented in the main article.

For VALOR the simulated genome was uniformly sampled using simulated fosmid clones ($\mu = 40Kbp$,$\sigma = 10Kbp$) at 5X coverage into 300 pools. The reads for the pools were simulated again using ART at 10X coverage. We employed VALOR by setting parameters min and max inversion size to 40Kbp and 10Mbp. 24 inversion breakpoints were by chance not covered by any clone thus remained undetectable by VALOR.

The sensitivity and precision was calculated at each cut off (inversion size > cut off) to illustrate how the performance of valor increases for detecting larger inversions. We calculated the overlap for inversions using the intersectBed tool in the BEDtools 2.26 suite. Supplementary Figures 10, 11, 12, and 13 show the performance of each tool for inversion greater than some cutoff at 50% and 90% reciprocal overlap. As the figures demonstrate, LUMPY is very sensitive to read length (compared to the 150bp long reads in the main article) and DELLY2 has better performance in smaller inversions. The inversions marked as precise by DELLY2 have are shown separately. At large inversions VALOR outperforms both tools. All the files output by the tools and the variations implanted are in Additional File 2.

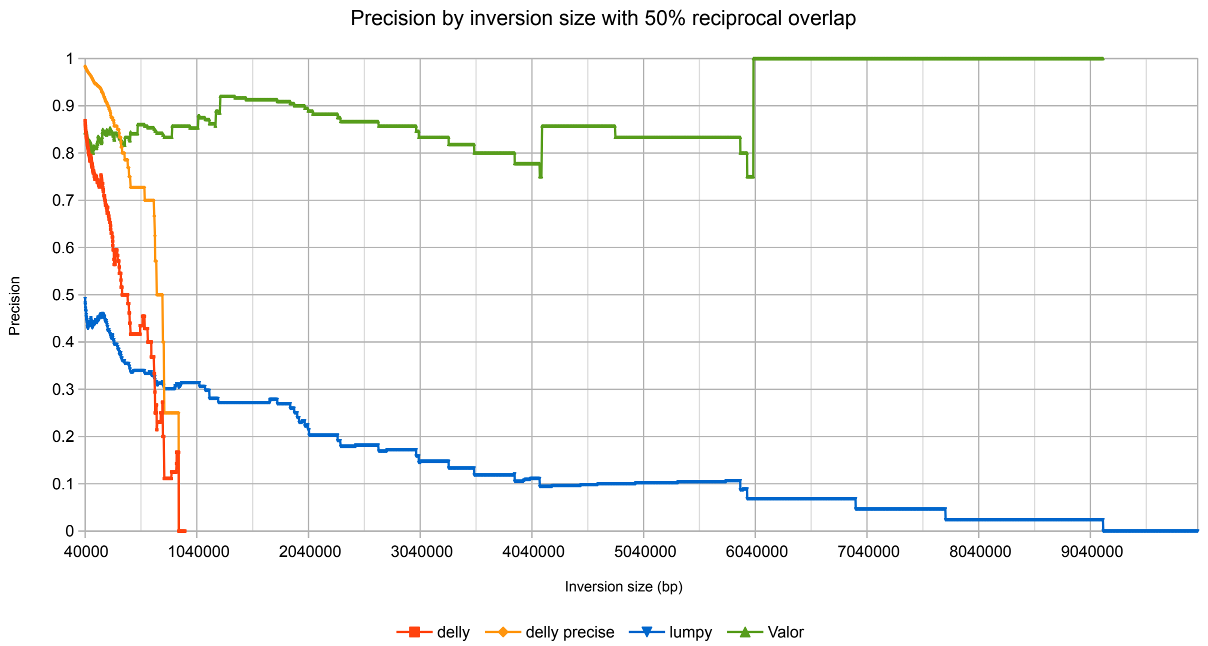

Supplementary Figure 10: Precision of WGS tools DELLY2 and LUMPY compared to Valor on VarSim simulated data at 50% reciprocal intersection with BEDtools.

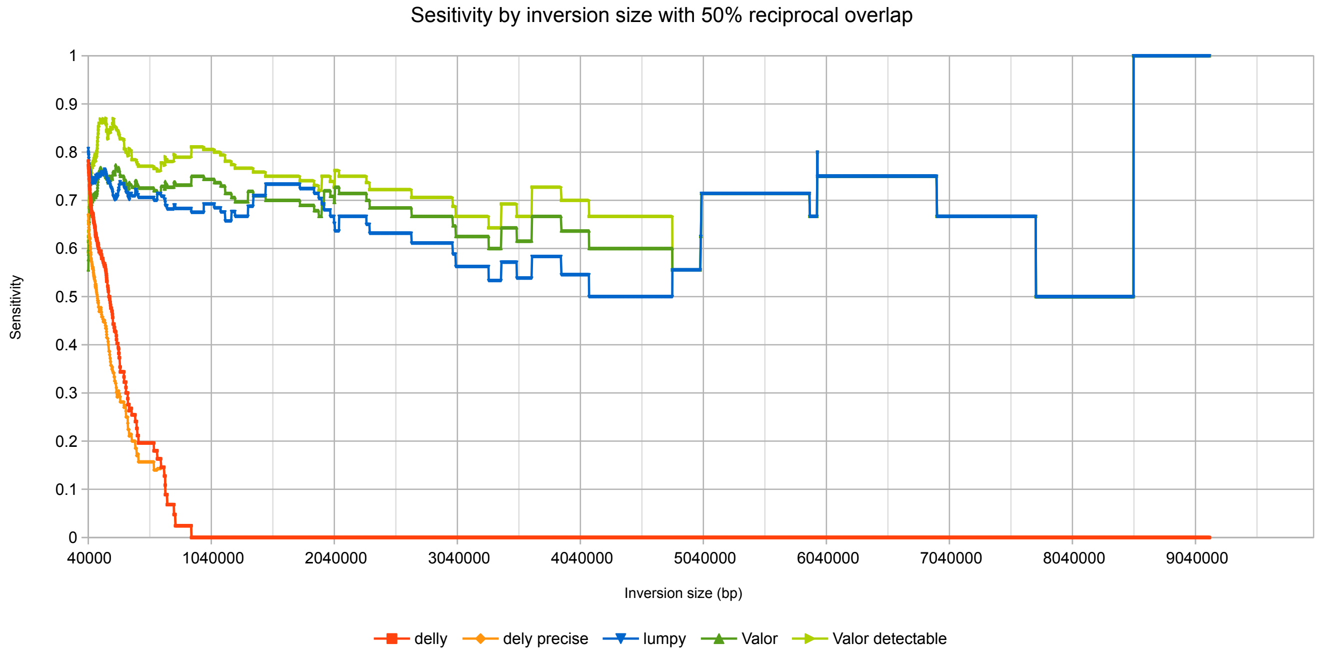

Supplementary Figure 11: Sensitivity of WGS tools DELLY2 and LUMPY compared to Valor on VarSim simulated data at 50% reciprocal intersection with BEDtools.

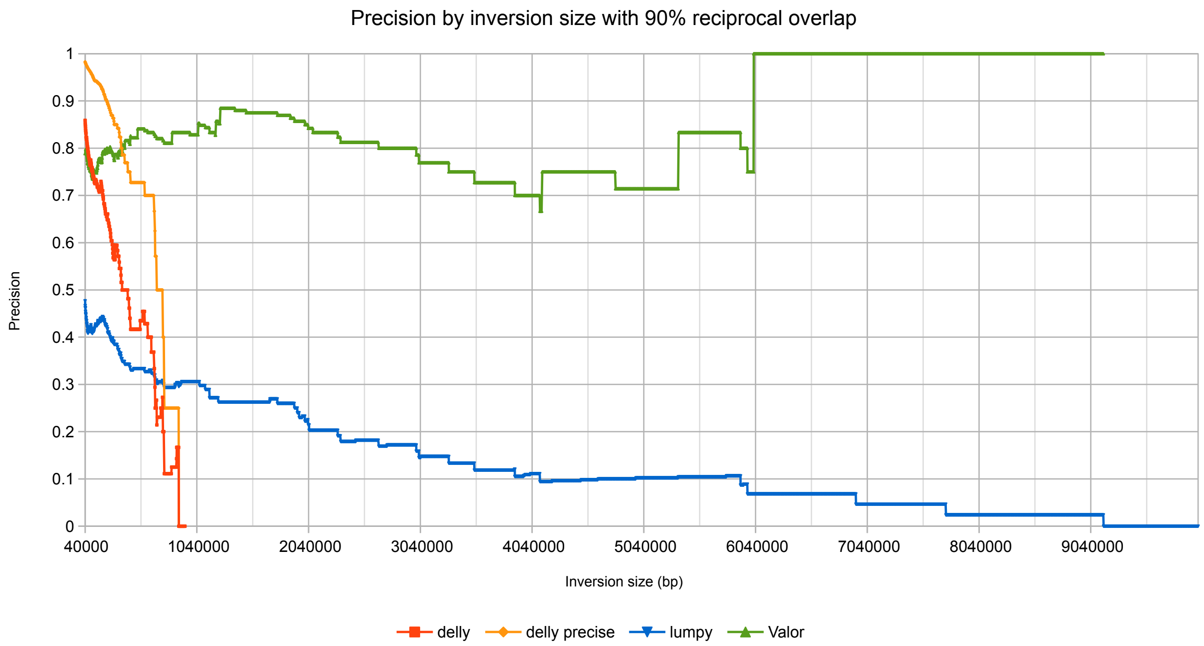

Supplementary Figure 12: Performance of WGS tools DELLY2 and LUMPY compared to VALOR on VarSim simulated data at 90% reciprocal intersection with BEDtools.

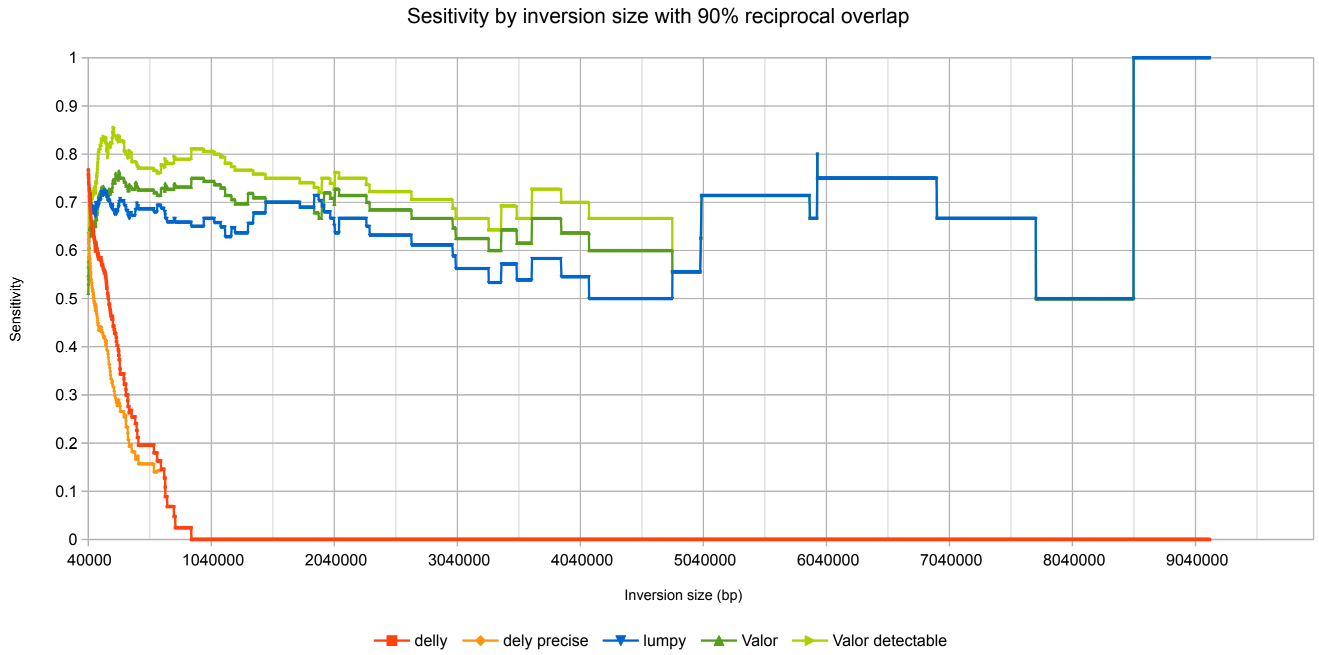

Supplementary Figure 13: Performance of WGS tools DELLY2 and LUMPY compared to VALOR on VarSim simulated data at 90% reciprocal intersection with BEDtools.

1.15 Statistics on the real data of the NA12878 individual

After simulations, Valor was applied to the pooled clone data from the genome of the NA12878 individual. Some statistics on the data are given bellow.

Supplementary Table 30: Number and percentage of mapping paired-end reads before and after removing duplicated ones

Set	Before	After	Distinct	Duplicated
**set1**	382,782,082	324,302,909	84.72%	15.28%
**set2**	223,707,355	190,888,484	85.33%	14.67%
**set3**	420,380,434	383,907,969	91.32%	8.68%
**ALL**	102,686,9871	899,099,362	87.56%	12.44%

Supplementary Table 31: Average number of normal size clones (125 Kbp-175 Kbp) inferred for each pool in each set vs. the expected number of clones

Clones	set1	set2	set3
**Expected**	230	389	153
**With 0s**	162	238	76
**Without 0s**	179	305	152

With 0 included the pools that had no inferred clones at all. Assuming that those probes might have been problematic, we also give the average numbers without including pools with zero clones as *Without 0*.
The difference is due to error or split clones.

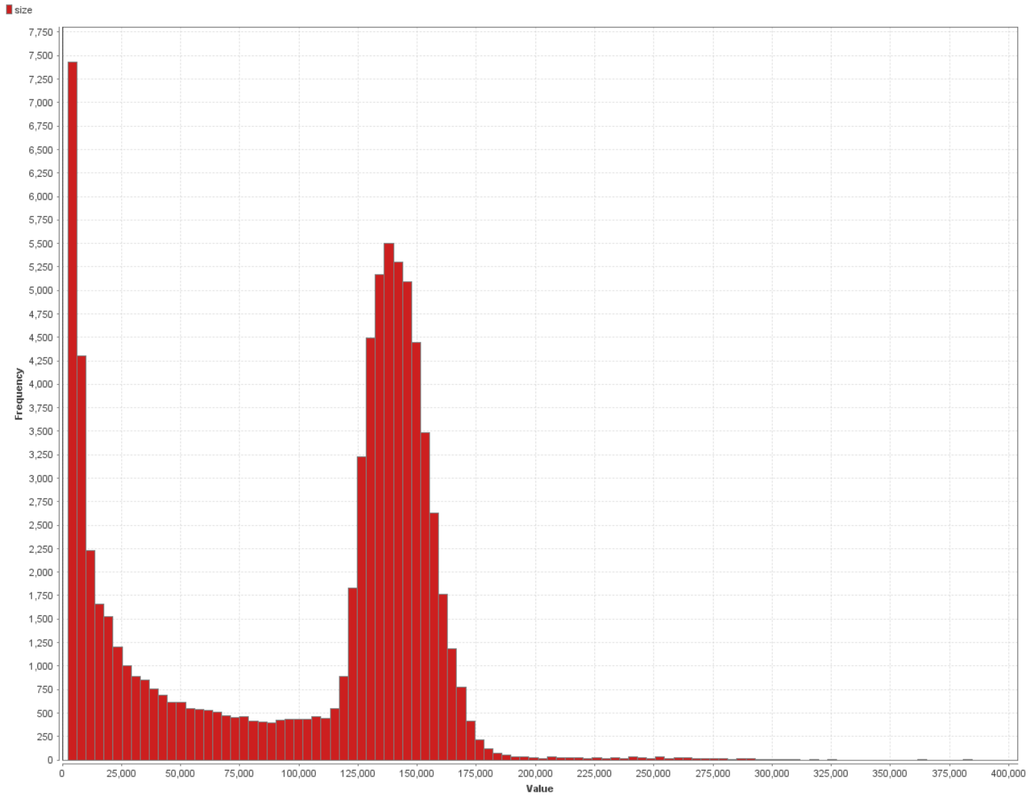

Supplementary Figure 14: Histogram of inferred clone size with 100 bins

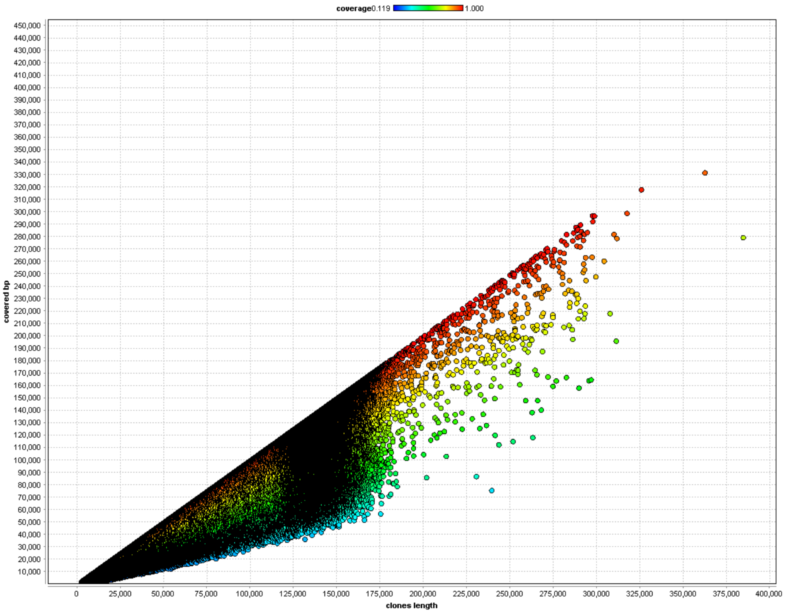

Supplementary Figure 15: Scatter plot of covered bp over clone size colored by coverage rate: It can be observed that clones of average size or larger are better covered

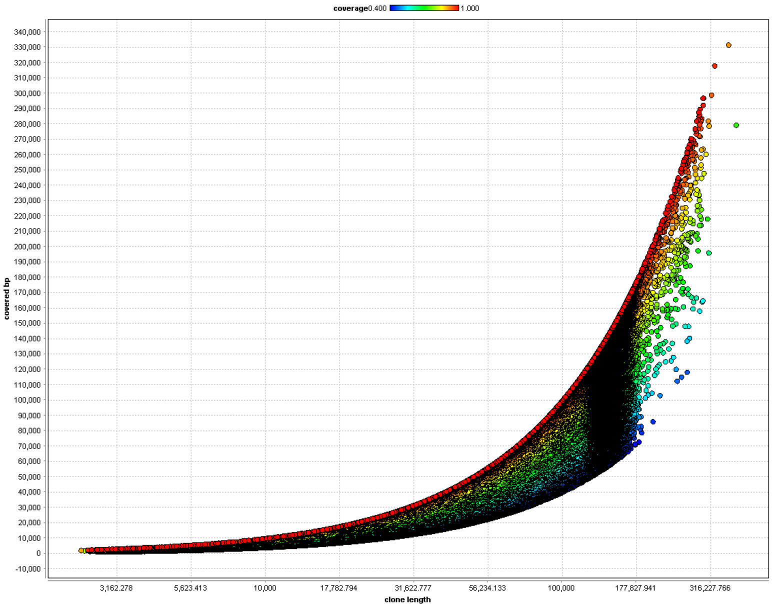

Supplementary Figure 16: Scatter plot of covered bp over log of clone length colored by coverage rate with cutoff of 40% coverage: It can be observed that clones of average size or larger are better covered

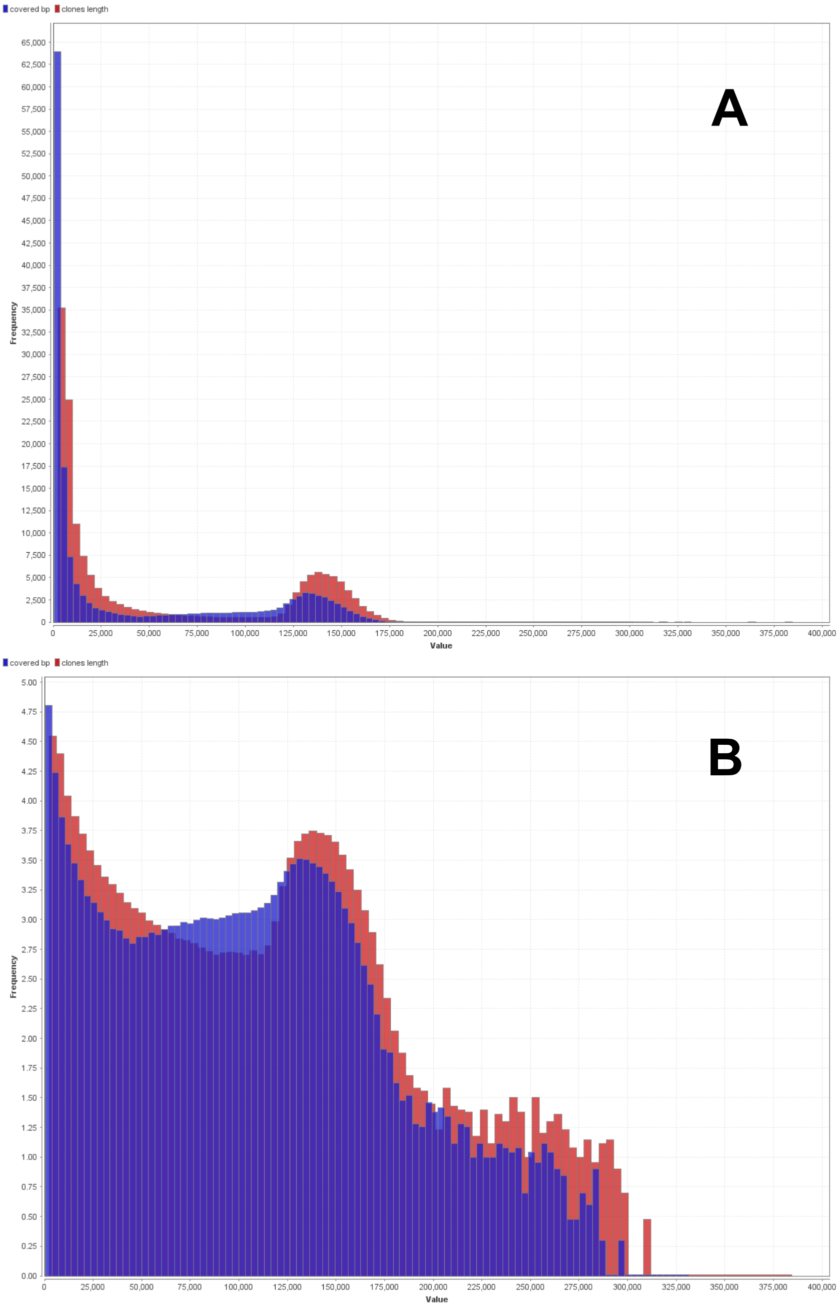

Supplementary Figure 17: (A) Histogram of covered bp over clone length with 100 bins and (B) Histogram of log of covered bp over log of clone length with 100 bins

1.16 Inversions predicted on the real data of the NA12878 individual

On the pooled BAC clones from the NA12878 genome, we aligned the paired-end reads using BWA-MEM and ran Valor with the parameters (min and max inversions) set to 100 Kbp and 10 Mbp. After running Valor and obtaining the clusters, we removed clusters with cluster size less than 2. The predicted inversion clusters are given in the Supplementary Table 32. For the sake of readability, we have assigned unique IDs to the inversions detected by Valor (in coordinate-sorted order). AB and CD are the cluster sizes of split clones (see Supplementary figure 7) and pp and mm are the total reads supporting the inversion signature on the given breakpoints. The size is calculated as the difference of the outer coordinates. The confirmed inversions are given in bold font.

We also tried to lift the predicted inversion coordinated to hg18 to compare with InvFEST [8] using UCSC liftover tool[3]. The comparison is given in Supplementary Table 33. The lifted coordinates are given along with the InvFEST ID and the *status*, and finally the last column represents the *value* (where it was true or false). The *status* can be predicted or unreliable prediction, which InvFEST sets according to type of the prediction performed, or validated, which means it has been validated experimentally on the genome.

[3]https://genome.ucsc.edu/cgi-bin/hgLiftOver

Supplementary Table 32: VALOR call set on pooled clone sequencing data of the NA12878 individual

ID	chrom	left start	left end	right start	right end	AC	BD	pp	mm	size
dS1	chr1	13,124,363	13,142,580	13,638,653	13,639,171	1	2	1	2	514,808
dS2	chr1	16,845,976	16,923,473	17,125,650	17,192,698	3	7	2	7	346,722
dS3	chr1	142,670,981	142,673,939	143,163,791	143,170,513	5	10	9	5	499,532
dS4	chr1	143,676,334	143,702,036	147,849,843	147,882,344	5	3	22	29	4,206,010
dS5	chr1	144,700,347	144,710,164	144,960,069	144,976,473	2	4	4	4	276,126
dS6	chr1	144,830,507	144,834,248	146,471,573	146,478,344	2	1	1	2	1,647,837
dS7	chr1	146,002,094	146,006,716	147,489,038	147,548,595	1	2	4	5	1,546,501
dS8	chr1	146,443,133	146,449,819	148,300,929	148,304,686	14	2	1	4	1,861,553
dS9	chr1	149,364,330	149,371,254	149,749,621	149,756,945	22	16	15	10	392,615
dS10	chr2	90,471,094	90,486,252	91,692,053	91,759,768	3	1	4	3	1,288,674
dS11	chr4	49,132,791	49,149,263	49,633,433	49,642,387	70	78	129	175	509,596
dS12	chr5	68,874,516	68,915,972	69,729,710	69,771,119	2	26	15	7	896,603
dS13	chr5	69,113,005	69,196,224	70,030,035	70,067,890	2	3	20	6	954,885
dS14	chr7	57,690,113	57,696,922	57,884,934	57,892,632	1	3	1	5	202,519
dS15	chr7	61,163,477	61,215,788	61,431,150	61,433,283	1	2	4	7	269,806
dS16	chr7	64,603,898	64,651,022	64,998,550	65,065,573	1	3	7	5	461,675
dS17	chr7	72,515,193	72,533,307	74,308,848	74,342,804	1	52	5	3	1,827,611
dS18	chr7	76,161,926	76,165,992	76,600,868	76,680,815	5	1	1	1	518,889
**dS19**	**chr8**	**6,977,308**	**7,070,893**	**12,442,860**	**12,449,094**	**6**	**4**	**6**	**3**	**5,471,786**
dS20	chr8	7,453,968	7,524,896	7,901,867	7,906,817	2	1	3	1	452,849
dS21	chr8	11,968,472	12,065,748	12,214,705	12,308,281	4	3	4	3	339,809
dS22	chr9	39,396,867	39,397,779	39,926,408	39,927,022	3	1	1	3	530,155
dS23	chr9	39,610,566	39,619,240	47,034,631	47,035,047	2	2	5	3	7,424,481
dS24	chr9	39,846,127	39,877,872	41,461,480	41,492,209	1	2	5	1	1,646,082
dS25	chr9	41,095,170	41,193,239	43,453,524	43,511,328	1	2	4	1	2,416,158
dS26	chr9	41,969,157	41,980,937	44,224,992	44,277,305	1	5	3	3	2,308,148
dS27	chr9	44,995,173	44,999,871	45,727,935	45,728,840	3	12	7	23	733,667
dS28	chr9	45,736,367	45,753,162	46,090,666	46,139,443	3	5	5	2	403,076
dS29	chr9	66,717,901	66,742,433	69,897,210	69,921,583	8	11	14	20	3,203,682
dS30	chr9	69,690,071	69,747,623	70,378,781	70,388,661	1	5	1	2	698,590
dS31	chr10	17,837,717	17,892,080	18,084,807	18,139,005	8	3	2	7	301,288
dS32	chr10	46,627,777	46,682,629	48,855,724	48,871,218	1	2	6	6	2,243,441
dS33	chr14	19,427,037	19,429,945	20,129,457	20,134,420	4	5	4	8	707,383
dS34	chr15	20,305,613	20,311,845	21,285,111	21,318,365	3	5	6	8	1,012,752
**dS35**	**chr15**	**30,727,982**	**30,823,312**	**32,859,062**	**32,864,779**	**1**	**2**	**17**	**8**	**2,136,797**
dS36	chr16	14,948,016	15,030,478	16,396,932	16,472,694	8	6	8	15	1,524,678
**dS37**	**chr16**	**16,704,632**	**16,722,093**	**18,732,305**	**18,759,138**	**2**	**2**	**14**	**10**	**2,054,506**
dS38	chr16	21,461,477	21,482,090	21,893,378	21,913,853	34	12	17	17	452,376
dS39	chr16	21,853,565	21,863,712	30,241,620	30,251,974	2	17	11	4	8,398,409
dS40	chr16	32,663,146	32,672,648	33,236,085	33,270,553	13	14	46	5	607,407
**dS41**	**chr17**	**34,508,335**	**34,572,064**	**36,296,916**	**36,330,960**	**3**	**2**	**16**	**21**	**1,822,625**
dS42	chr17	44,372,193	44,400,349	44,577,375	44,618,085	14	1	9	11	245,892
dS43	chrX	52,117,948	52,191,606	52,395,583	52,466,291	1	4	1	1	348,343

The callset of VALOR on the total 288 pools of PCS data of the NA12878 genome. Parameters (min and max inversion size) were set to 100 Kbp and 10 Mbp. VALOR outputs 2 coordinates for each breakpoint. AB: the cluster size of the AC split clones. CD: the cluster size of the BD split clones. pp: number of paired-reads mapping on the same strand (direct) supporting the breakpoint intervals. mm: number of paired-reads mapping to the same strand (reverse) supporting the breakpoint intervals. size: the difference of the outer coordinates of the inversion. The inversions with cluster size < 2 were filtered out due to low quality of the data. Inversions in bold are the confirmed ones.

Supplementary Table 33: Comparison of inversions called by VALOR on the NA12878 genome to InvFEST database

ID	chrom	lifted start	lifted end	InvFEST ID	status	value
dS12	chr5	68,910,272	69,806,875			
dS13	chr5	69,148,761	70,103,646	HsInv0690	P	0
dS14	chr7	57,694,055	57,896,574			
dS16	chr7	64,241,333	64,703,008	HsInv0299	P	0
dS17	chr7	72,153,129	73,980,740			
dS18	chr7	75,999,862	76,518,751			
dS19	chr8	6,964,718	12,493,465	HsInv0501	V	1
dS33	chr14	18,497,037	19,204,260	HsInv0537, HsInv0761, HsInv0765	P, U, U	0, 0, 0
dS34	chr15	18,565,627	19,583,024	HsInv0770	U	0
dS35	chr15	28,515,274	30,652,071	HsInv1049	V	1
dS36	chr16	14,855,517	16,380,195	HsInv0365, HsInv0551, HsInv0780	U, U, P	0, 1, 1/0
dS37	chr16	16,612,133	18,666,639	HsInv0362, HsInv0368, HsInv0369, HsInv0560	U, P, U, P	0, 0, 0, 0
dS38	chr16	21,368,978	21,821,354			
dS39	chr16	21,761,066	30,159,475			
dS40	chr16	32,570,647	33,178,054			
dS41	chr17	31,532,448	33,534,539	HsInv1048	V	0, 0, 0
dS42	chr17	41,727,970	41,973,401			

Inversions predicted in Supplementary Table 32 were lifted to hg18 coordinated in order to look them up in InvFEST database. 17 of them could be lifted with $\geq$ 95% precision. The lifted outer coordinates are given in column 2 and 3, the InvFEST ID is given in the fourth column. Column status is the InvFEST status (P: predicted, U: unreliable prediction, V :validated). Column value shows whether the inversion was predicted/validates on the NA12878 genome (0: false, 1: true). In case of several InvFEST entries, they have been separated by commas. Value x/y means the prediction was done on each breakpoint separately (x:value on left breakpoint, y: value on right breakpoint).
* HsInv0496, HsInv0497, HsInv0712, and HsInv0713 also correspond to inversion dS19, but because their coordinates were duplicates, we excluded them.

17 inversions could be lifted with $\geq$ 95% precision, out of which 9 were overlapping $\geq$ 50% on breakpoints with 16 InvFEST inversions, excluding dS19 inversion which is repeated 4 more times with the same coordinates. Another mistake is that InvFEST indicated inversion dS41 has been validated to be STD/STD but Antonacci et. al. (2009) have validated it in their wok.

1.17 FISH validations

We chose a set of the predicted inversions and tried to validate the with FISH experiments.

Supplementary Table 34: Summary of FISH results on inversions predicted in the genome of NA12878 using dipSeq.

**ID**	**chrom**	**start**	**end**	**size**	**result**
ds13	chr5	69,080,890	70,004,538	4,214,658	*not tested*
ds33	chr14	19,369,507	20,154,427	1,336,417	*not tested*
ds37	chr16	16,722,093	18,732,305	2,010,212	confirmed
ds39	chr16	21,847,556	30,283,910	883,731	*not confirmed*
ds40	chr16	32,277,947	33,295,746	1,090,400	*not tested*

not tested: The inversion was not tested because they were located on Segmental Duplications.

1.18 Extra files

The comparison results mentioned in the paper can be found under the supplementary folder. The genomes studied are grouped into sub folders:

- **CHM1** – 11 files
- **NA12877** – 6 files
- **NA12878** – 12 files
- **NA12882** – 6 files
- **simulation/varsim1** – 6 files
- **simulation/varsim2** – 4 files

In each case the format is: $[tool]_{[}genome]_{[}reference].[fileformat]$

Tools are DELLY2, LUMPY, and GASVPro. In case of the CHM1 genome, the reads were mapped to both GRCh37 reference and the CHM1 assembly. In the rest of the data, the reference is GRCh37. GASVPro outputs 4 breakpoints for each inversion, the files suffixed with *inner* use the inner breakpoints and *outer* give the outer breakpoints to create bed formats. bed formats are required to compare against the truth set.

For CHM1 there is a truth set of inversions tested by the authors which we downloaded and is available in *inversions_truth.bed* file.

In case of NA12878, we examined two data sets, one older PCR data with lower quality, and another with higher quality and PCR-free.

In all cases reads were mapped by BWA-MEM to the reference, duplicate reads were removed with Picard, and the remaining were realigned around indels with GATK.

References

[1] Li, H., Durbin, R.: Fast and accurate short read alignment with Burrows-Wheeler transform. Bioinformatics **25**(14), 1754–1760 (2009). doi:10.1093/bioinformatics/btp324

[2] Xin, H., Lee, D., Hormozdiari, F., Yedkar, S., Mutlu, O., Alkan, C.: Accelerating read mapping with FastHASH. BMC Genomics **14 Suppl 1**, 13 (2013). doi:10.1186/1471-2164-14-S1-S13

[3] Brunato, M., Hoos, H.H., Battiti, R.: In: Maniezzo, V., Battiti, R., Watson, J.-P. (eds.) On Effectively Finding Maximal Quasi-cliques in Graphs, pp. 41–55. Springer, Berlin, Heidelberg (2008). doi:10.1007/978-3-540-92695-5_4. http://dx.doi.org/10.1007/978-3-540-92695-5_4

[4] Hormozdiari, F., Alkan, C., Eichler, E.E., Sahinalp, S.C.: Combinatorial algorithms for structural variation detection in high-throughput sequenced genomes. Genome Res **19**(7), 1270–1278 (2009). doi:10.1101/gr.088633.108

[5] Fiume, M., Smith, E.J.M., Brook, A., Strbenac, D., Turner, B., Mezlini, A.M., Robinson, M.D., Wodak, S.J., Brudno, M.: Savant genome browser 2: visualization and analysis for population-scale genomics. Nucleic Acids Res **40**(Web Server issue), 615–621 (2012). doi:10.1093/nar/gks427

[6] Layer, R.M., Chiang, C., Quinlan, A.R., Hall, I.M.: LUMPY: a probabilistic framework for structural variant discovery. Genome Biol **15**(6), 84 (2014). doi:10.1186/gb-2014-15-6-r84

[7] Rausch, T., Zichner, T., Schlattl, A., Stütz, A.M., Benes, V., Korbel, J.O.: DELLY: structural variant discovery by integrated paired-end and split-read analysis. Bioinformatics **28**(18), 333–339 (2012). doi:10.1093/bioinformatics/bts378

[8] Martínez-Fundichely, A., Casillas, S., Egea, R., Ràmia, M., Barbadilla, A., Pantano, L., Puig, M., Cáceres, M.: InvFEST, a database integrating information of polymorphic inversions in the human genome. Nucleic Acids Res **42**(Database issue), 1027–1032 (2014). doi:10.1093/nar/gkt1122
